# Evolution of macromolecular crystallography beamlines at the Swiss Light Source and SwissFEL

**DOI:** 10.1107/S1600577525005016

**Published:** 2025-07-14

**Authors:** Meitian Wang

**Affiliations:** a Swiss Light Source, Center for Photon Science Paul Scherrer Institute Forschungsstrasse 111 5232Villigen Switzerland; Cornell University, USA

**Keywords:** macromolecular crystallography, synchrotron beamline, Swiss Light Source 2.0, structural biology, structural dynamics

## Abstract

This review highlights the development and evolution of three macromolecular crystallography (MX) beamlines at the Swiss Light Source (SLS) over the past two decades. We discuss key advancements in X-ray optics, detectors, goniometers, sample changers and MX methodology, emphasizing their impact on high-throughput and high-resolution structural biology. Our contributions are presented within the broader context of global efforts in synchrotron-based MX. Looking ahead, we explore the future experiments enabled by SLS 2.0 and new opportunities at SwissFEL to enhance experimental capabilities and drive scientific discoveries.

## Abstract 2

This review examines two decades of continuous development in macromolecular crystallography beamlines at the Swiss Light Source, along with recent contributions from SwissFEL, in the broader context of the evolution of macromolecular crystallography at synchrotron and X-ray free-electron laser facilities.

## 1. Introduction

Following the success of high-energy third-generation synchrotrons in the 1990s – such as the 6 GeV European Synchrotron Radiation Facility—ESRF (1994), 7 GeV Advanced Photon Source—APS (1995) and 8 GeV Super Photon Ring—SPring-8 (1997) – the Swiss Light Source (SLS) started user operation in 2001 as a medium-energy synchrotron (2.4 GeV) (Nolting *et al.*, 2023 ▸). The SLS features a compact design with 288 m circumference, where the booster and storage rings share the same tunnel. The source emittance is 5500 pm rad and 5 pm rad (horizontal versus vertical). From the start, the SLS operated in top-up mode (Böge, 2002 ▸), ensuring stable, continuous beamline operation with a 400 mA electron beam current (Ludeke *et al.*, 2006 ▸). The success of the SLS marked the beginning of a new era, leading a wave of new national synchrotron facilities around the world (https://lightsources.org/).

The SLS supports various scientific applications across 18 beamlines, with three being dedicated to macromolecular crystallography (MX) (Hendrickson, 2000 ▸; Helliwell, 1992 ▸). In 2024, the MX beamlines celebrated 10000 structures in the Protein Data Bank (PDB) and 4700 publications (Fig. S1 of the supporting information), which count for half of all publications from the SLS. In addition, numerous structures have been determined for proprietary research in drug discovery (Hennig *et al.*, 2012 ▸; Tosstorff *et al.*, 2022 ▸; Vulpetti *et al.*, 2023 ▸; Käck & Sjögren, 2025 ▸). The Swiss Free-Electron Laser (SwissFEL) was established in 2017 to complement the SLS. SwissFEL has two branches to cover both hard X-ray and soft X-ray applications, and two endstations provide capabilities for serial femtosecond crystallography (SFX) with a variety of sample delivery methods (Milne *et al.*, 2017 ▸; Nolting *et al.*, 2023 ▸).

## 2. Three MX beamlines at the SLS – X06SA-PXI, X10SA-PXII and X06DA-PXIII

The first MX beamline X06SA-PXI was designed to leverage in-vacuum undulator technology (Hara *et al.*, 1998 ▸), extending high-brightness radiation into the hard X-ray regime (3–18 keV) within a medium-energy synchrotron. This achievement was made possible through a collaboration between the Paul Scherrer Institute (PSI) and SPring-8, which facilitated the installation and operation of the first in-vacuum undulator (U24) at X06SA-PXI shortly after the SLS storage ring’s commissioning in 2001 (Ingold *et al.*, 2007 ▸). The in-vacuum, small-gap, short-period undulator with small phase error operating on higher harmonics proved to be a stable source of high-brightness radiation, leading to the installation of similar undulators at other SLS beamlines, including two U19 undulators at X06SA-PXI (replacing U24) and X10SA-PXII.

The X06SA-PXI source size and divergence are 202 µm × 23 µm and 135 µrad × 25 µrad (horizontal versus vertical, FWHM) at 12.4 keV, respectively. The X-ray optics system was engineered to maximize the flux by collecting the full undulator harmonics while enabling adaptive control of the beam size and divergence at two sample positions. This was achieved by combining a novel double-crystal monochromator featuring a sagittal bender on the second crystal (Schulze-Briese *et al.*, 1998 ▸) and a flexural hinge-based mirror bender in the vertical focusing mirror (VFM) (Rossetti *et al.*, 2002 ▸). The sagittally bendable Si crystal collected up to 150 µrad of X-rays and focused them in the horizontal direction while the VFM provided achromatic focusing in the vertical direction with two stripes of different surface material, uncoated Si and Rh. The beamline was fully tunable between 5.7 and 17.5 keV and was optimized for energies around the selenium *K*-edge of 12.66 keV with an energy resolution of 2 × 10^−4^ for experimental phasing using multiple- and single-wavelength anomalous diffraction (MAD/SAD). At the starting time of the beamline, about half of the structures needed *de novo* phasing (Hendrickson, 2014 ▸). With the brighter X-ray beam, control of radiation damage became an integral part of the MX data collection and crucial in experimental phasing. To this end, we introduced absolute flux and dose estimation (Owen, Holton *et al.*, 2009 ▸) to calculate data collection strategies (Dauter, 1999 ▸).

The beamline hosted two experimental endstations for high-resolution diffraction (HRD) and micro-diffraction (MD). The HRD station, optimized for resolving crystals with large unit cells, provided a low-divergence beam (85 µm × 10 µm, 320 µrad × 70 µrad) with a flux of 2 × 10^12^ photons s^−1^ at 12.4 keV. The beam size could be adjusted to match crystal size, enhancing the signal-to-noise ratio. Thanks to the minimal aberration in sagittal focusing and low slope error of the VFM, at the MD station designed for micro-crystallography, tight focusing (25 µm × 5 µm, 1100 µrad × 350 µrad) with the same flux was achieved. Horizontal beam size could be further reduced to 10 or 5 µm using apertures integrated with a micro-diffractometer developed at the European Molecular Biology Laboratory (EMBL, Grenoble) (Perrakis, Cipriani *et al.*, 1999 ▸). A secondary source and Kirkpatrick–Baez (KB) mirror system were added in 2012 to enhance flexibility, allowing finer control over the beam size and divergence at the sample position. The beam size range 1–100 µm addressed the user community’s demand nicely (Fig. S2 of the supporting information).

The second beamline X10SA-PXII was constructed for proprietary research exclusively with the three founding partners, Max-Planck-Gesellschaft, Novartis and Roche, in 2004 (Diez *et al.*, 2007 ▸). The X-ray optics design was built based on the experience of X06SA-PXI, leading to a focused beam size and divergence of 50 µm × 10 µm and 540 µrad × 130 µrad at 12.4 keV, respectively. The energy range was extended to 20 keV with an additional Pt coating on the vertical focusing mirror. A dedicated diffractometer (D3) (Fuchs *et al.*, 2014 ▸) was developed with three key features: (1) sub-micrometre sphere of confusion for the horizontal single-axis rotation and fast sample-rastering; (2) beam-shaping apertures for micro-crystallography with a 10 µm beam; (3) on-axis microspectrophotometer for multi-mode optical spectroscopy (Owen, Pearson *et al.*, 2009 ▸; Pompidor *et al.*, 2013 ▸). Later, a secondary source and kinoform lenses were added to explore refraction-based X-ray focusing for MX beamlines (Lebugle *et al.*, 2018 ▸). The beamline served academic research and structure-based drug discovery, and gradually attracted more industry partners. Part of the ‘in-house research’ beam time was also used for the Structural Genomics Consortium (SGC) (Williamson, 2000 ▸) before the Diamond Light Source (DLS) was constructed.

The third beamline, X06DA-PXIII, exploited the small electron beam size at a bending magnet source and used a 2.7 T super-bending magnet to push the critical energy to 11 keV for MX applications. The X-ray focusing optics consisted of a vertical collimating mirror in the front end and a toroidal focusing mirror. The focusing aberration of the toroidal mirror was minimized using a 2:1 focusing ratio (MacDowell *et al.*, 2004 ▸), which produced a fixed focus of 90 µm × 45 µm with divergence of 2 mrad × 0.5 mrad at the sample position. One unique design was the double channel-cut monochromator for a true fixed-exit of X-rays for the 5–17.5 keV energy range. Energy changes required only two rotations, ensuring both speed and stability. This design was later proven to be beneficial for experimental phasing.

The endstation was inspired by the mini-hutch design of beamline 8.3.1 from the Advanced Light Source (ALS) (MacDowell *et al.*, 2004 ▸). The experimental hutch had three access windows for different experimental modes. The front window enabled fast sample exchange in manual operation, the side window was used for loading pucks into the sample changer, and the back window, serving as a portal for fully automated *in situ* screening of crystallization plates (Fig. S3 of the supporting information), was connected to a crystallization facility. The latter was integrated at the beamline to facilitate on-site sample preparation, crystallization and *in situ* screening experiments (Bingel-Erlenmeyer *et al.*, 2011 ▸). The PSI and industry partners – Actelion (now Idorsia), Boehringer Ingelheim, Mitsubishi Chemical, Novartis and Proteros Biostructures – co-financed the beamline. X06DA-PXIII started user operation in 2008.

**Figure 1:**
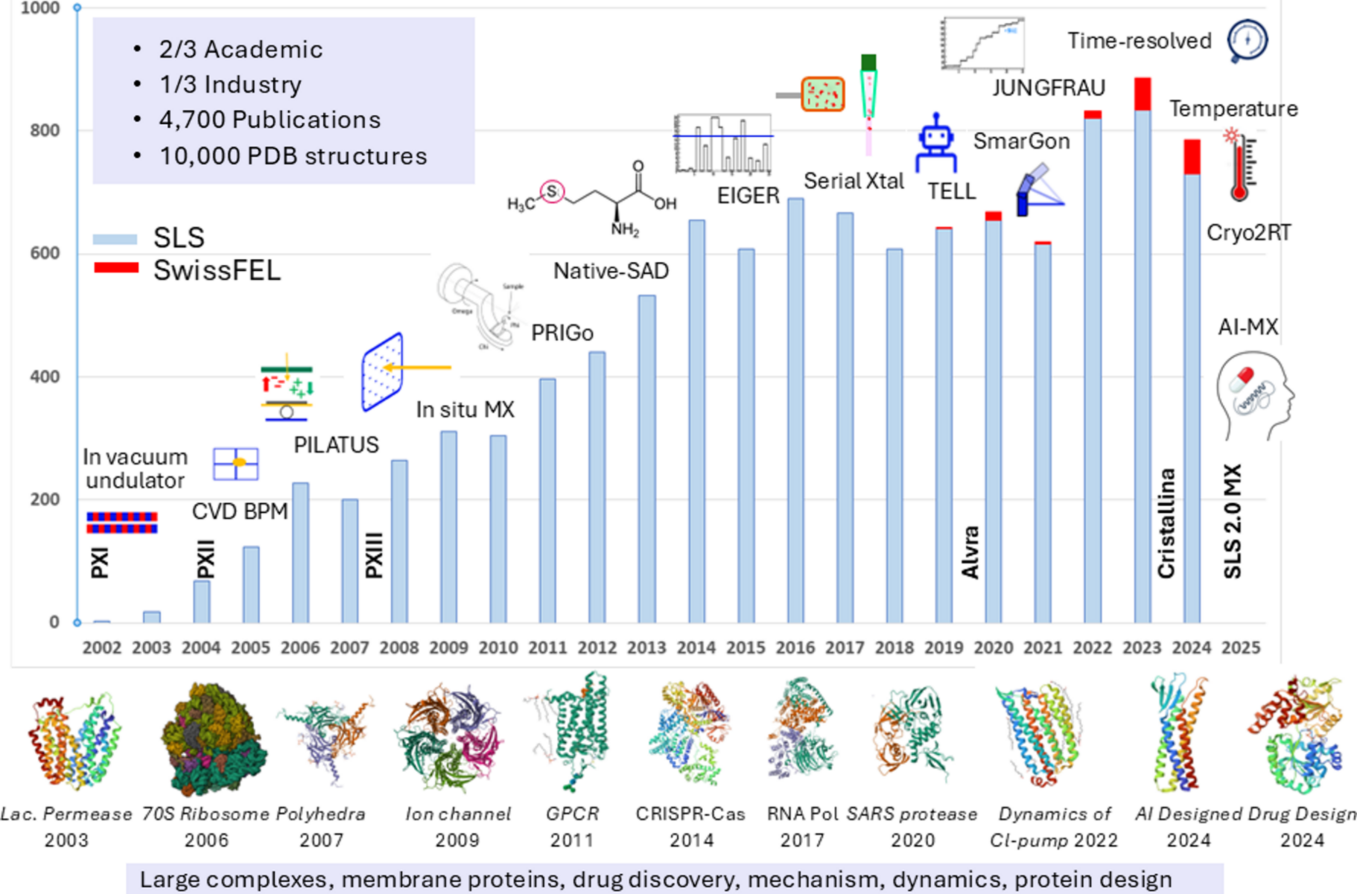
Annual PDB depositions (SLS – light blue bar, SwissFEL – red bar), development of MX beamlines at SLS and SwissFEL and selected scientific highlights [Lac. Permease (PDB entry 1pv6; Abramson *et al.*, 2003 ▸), 70S Ribosome (PDB entry 4v51; Selmer *et al.*, 2006 ▸), polyhedra (PDB entry 2oh5; Coulibaly *et al.*, 2007 ▸), ion channel (PDB entry 3ehz; Hilf & Dutzler, 2009 ▸), GPCR (PDB entry 2x72; Standfuss *et al.*, 2011 ▸), CRISPR-Cas (PDB entry 4cmp; Jinek *et al.*, 2014 ▸; Yamano *et al.*, 2016 ▸), RNA polymerase (PDB entry 5o7x; Engel *et al.*, 2017 ▸), SARS protease (PDB entry 6yva; Shin *et al.*, 2020 ▸), Chloride-pump (PDB entry 7o8y; Mous *et al.*, 2022 ▸), designed GPCR (PDB entry 8oyv; Goverde *et al.*, 2024 ▸), Industry drug design (PDB entry 8pfp; Ferretti *et al.*, 2024 ▸)].

The three beamlines complemented each other and fostered the development of transformative MX techniques and methods. They are among the most productive MX beamlines worldwide (Zheng *et al.*, 2014 ▸; Grabowski *et al.*, 2021 ▸). The beamline development timeline and selected highlights are illustrated in Fig. 1 ▸. The main beamline characteristics and the X-ray optics design are listed in Table 1 ▸ and Table S1 of the supporting information.

## 3. Innovations in MX beamline technology and methods development

Driven by advancements and automation in protein production and crystallization, global structural genomics initiatives, and structure-based drug discovery, continuous innovation in MX beamline technology over the past three decades has transformed synchrotron MX. What was once a specialized technique has now become a widely accessible and indispensable tool for both academic research and industrial applications. The SLS has made a few valuable contributions to this global endeavor. Techniques developed for one specific application were often essential for other unforeseen or yet unknown applications. We highlight some of them here.

### 3.1. Integrated X-ray optics concept for reliable beamline operation

The X-ray optics system was designed to maintain a stable X-ray beam position across the entire energy range by integrating high-resolution mechanics, real-time beam position monitoring and active feedback. The mechanics of monochromator and mirror benders were engineered for precise beam steering with micrometre-level precision. Such precision is essential for MX experiments, where even minor misalignments can affect the accuracy of measurements and data quality.

However, maintaining a micrometre-sized beam at the sample position over extended periods is challenging due to potential drifts in the electron orbit and thermal fluctuations in the optics system, especially after changing the X-ray energy with the monochromator. To address these issues, we developed a quadrant X-ray position monitor chemical vapor deposition (CVD) diamond, capable of tracking the X-ray beam position with micrometre precision (Schulze-Briese *et al.*, 2001 ▸; Pradervand *et al.*, 2004 ▸). The 12 µm-thin CVD diamond is nearly transparent over the full energy range of the beamlines and multiple sensors can be installed along the X-ray path to monitor both beam position and angle. This system enables real-time correction of beam drift by steering the X-ray optics and automates energy changes without the need for manual intervention, thereby making beamline operation and MAD/SAD phasing experiments more user-friendly and boosting overall productivity. Additionally, this sensor technology has been commercialized by DECTRIS Ltd and made available to the synchrotron community.

### 3.2. X-ray detectors from PILATUS, EIGER to JUNGFRAU

**Figure 2:**
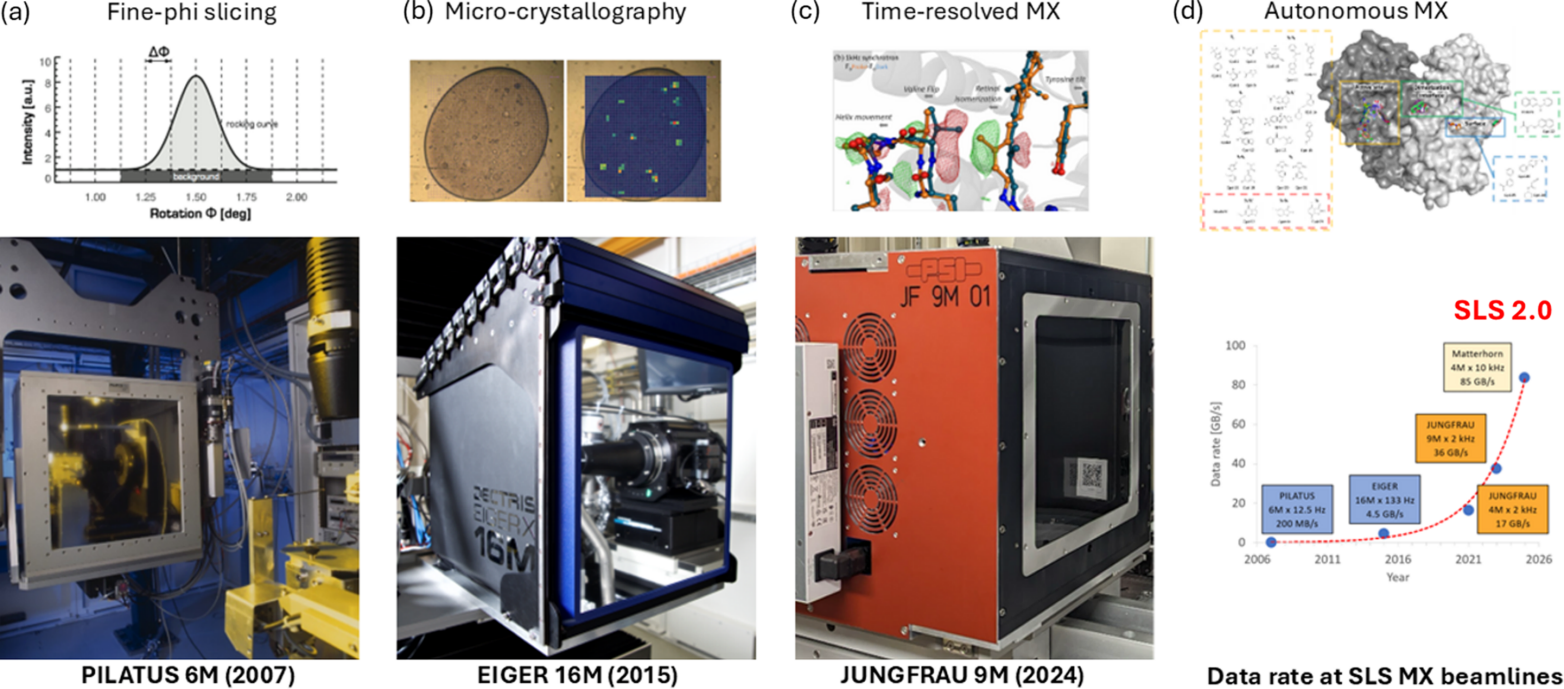
Detector evolution and selected applications. (*a*) The PILATUS detector enabled fine-phi slicing data collection (Mueller *et al.*, 2012 ▸). (*b*) The high frame-rate EIGER detector allowed fast 2D diffraction scan for micro-crystallography (Wojdyla *et al.*, 2016 ▸). (*c*) The kilohertz JUNGFRAU detector and millisecond time-resolved crystallography (Leonarski, Nan *et al.*, 2023 ▸). (*d*) Data rate growth and autonomous MX for ligand screening (Huang, Metz *et al.*, 2024 ▸).

For detecting the X-ray diffraction, large format charge-coupled device (CCD) detectors were developed and widely used at MX beamlines at third-generation synchrotrons from the 1990s (Strauss *et al.*, 1990 ▸; Gruner *et al.*, 2002 ▸). The SLS MX beamlines were initially also equipped with MAR165 and MAR225 CCD detectors (https://www.rayonix.com). To fully harness the increasing brightness at synchrotron beamlines, PSI initiated what was later referred to as PILATUS project to develop a large-format hybrid pixel-array detector for MX applications (Eikenberry *et al.*, 2003 ▸). The first generation of PILATUS detectors was commissioned at the SLS, with a PILATUS 6M introduced for user operation at X06SA-PXI in 2007 (Henrich *et al.*, 2009 ▸) [Fig. 2 ▸(*a*)]. Building on this innovation, the next-generation detector, EIGER, was launched in 2015, featuring smaller pixel sizes, shorter readout times and higher frame rates (Dinapoli *et al.*, 2011 ▸) [Fig. 2 ▸(*b*)].

The unique features of PILATUS/EIGER detectors transformed MX data collection and processing, profoundly impacting synchrotron MX (Förster *et al.*, 2019 ▸). Single-photon sensitivity, zero point-spread function and high dynamic range enabled precise recording of both weak and strong diffraction spots. This capability was especially critical for challenging experiments, such as crystallography of ribosomes and large molecular complexes (Fig. S4 of the supporting information) (Neubauer *et al.*, 2009 ▸), where the single-photon sensitivity and zero-readout noise allowed thousands of weak intensity reflections at high diffraction angles to be captured, reaching higher resolution from crystals with unit-cell dimensions as large as 1000 Å.

In addition, the deadtime-free millisecond readout time made continuous, shutterless data collection feasible, improving data collection precision and reducing data collection time. Moreover, the combination of zero readout noise and fine-phi slicing minimized background noise, enhancing the signal-to-noise ratio and enabling higher diffraction resolution (Mueller *et al.*, 2012 ▸; Casanas *et al.*, 2016 ▸; Pflugrath, 1999 ▸) [Fig. 2 ▸(*a*)]. This technological leap facilitated a paradigm shift in data collection strategies, moving away from high-dose, low-redundancy methods toward low-dose, high-redundancy approaches (Weinert *et al.*, 2015 ▸; Winter *et al.*, 2019 ▸). By the time PILATUS arrived, several automated data processing pipelines were available (Holton & Alber, 2004 ▸; Minor *et al.*, 2006 ▸). Still, none were optimized to match data processing time to data collection time, which was reduced to a few minutes with PILATUS. We exploited the parallel data processing of *XDS* (Kabsch, 2010*a* ▸; Kabsch, 2010*b* ▸) using high-performance computing clusters and developed the pipeline *go.com* (unpublished) with simple decision-making approaches. *go.com* provided fast feedback on key data processing results (*i.e.* diffraction resolution, completeness, *I*/σ, CC_1/2_, CC_ano_ and possible space groups) within a few minutes after data collection, proving essential for the era of high-throughput MX. Similar approaches were used elsewhere thereafter (Winter, 2010 ▸; Vonrhein *et al.*, 2011 ▸; Monaco *et al.*, 2013 ▸).

Furthermore, the high frame rates enabled fast and continuous grid scans for diffraction-based centering and micro-crystallography. In the past, diffraction cartography – used to identify optimal diffraction regions within crystals (Bowler *et al.*, 2010 ▸) or to locate microcrystals (Cherezov *et al.*, 2009 ▸) – was limited by CCD readout times and noise. Continuous grid scanning was first implemented at DLS using a PILATUS detector (12.5 Hz) (Aishima *et al.*, 2010 ▸), and faster protocols (100 Hz) were realized with the EIGER detector at SLS (Wojdyla *et al.*, 2016 ▸) [Fig. 2 ▸(*b*)]. These advancements were essential for the development of serial synchrotron crystallography (SSX) (Diederichs & Wang, 2017 ▸).

Commercialized and further advanced by DECTRIS Ltd, PILATUS and EIGER detectors are now integral to synchrotron facilities worldwide, significantly improving X-ray data quality and experimental throughput. This technology has played a key role in the exponential growth of protein structure determination over the past two decades, also providing essential datasets for training breakthrough tools like *AlphaFold2* (Jumper *et al.*, 2021 ▸).

To complement photon-counting detectors, the PSI’s detector group developed the JUNGFRAU charge-integrating pixel-array detector for applications in both X-ray free-electron laser (XFEL) and synchrotron environments (Mozzanica *et al.*, 2018 ▸) [Fig. 2 ▸(*c*)]. Its adaptive gain technology offers single-photon sensitivity and a high dynamic range without count-rate limitations. The JUNGFRAU detector is particularly well suited for MX applications at low energy and high flux due to its high dynamic range, low noise performance and fast readout speed. These features were key to handling high photon rates and improving data accuracy (Leonarski *et al.*, 2018 ▸; Chapman, 2018 ▸). While JUNGFRAU performs efficiently at the pulsed sources with moderate repetition rates of some XFELs, *e.g.* 100 Hz in the case of SwissFEL, achieving ‘continuous’ synchrotron operation requires kilohertz frame rates, presenting challenges in data throughput. To address this, the *Jungfraujoch* system was developed, capable of handling 38 GB s^−1^ on a single server using field-programmable gate arrays (FPGAs) and general-purpose GPUs (Leonarski *et al.*, 2020 ▸; Leonarski, Brückner *et al.*, 2023 ▸). Additionally, *Jungfraujoch* integrates basic crystallographic data analysis, including background integration, spot finding and indexing (Gasparotto *et al.*, 2024 ▸), enabling real-time monitoring, analysis and feedback at kilohertz frame rates.

All in all, continuous advancements in X-ray detectors have played an instrumental role in boosting beamline productivity and data quality, enabling new MX applications, including autonomous experiments and driving an exponential increase in data rates [Fig. 2 ▸(*d*)] (Leonarski, Brückner *et al.*, 2023 ▸).

### 3.3. From single-axis to multi-axis goniometer

**Figure 3:**
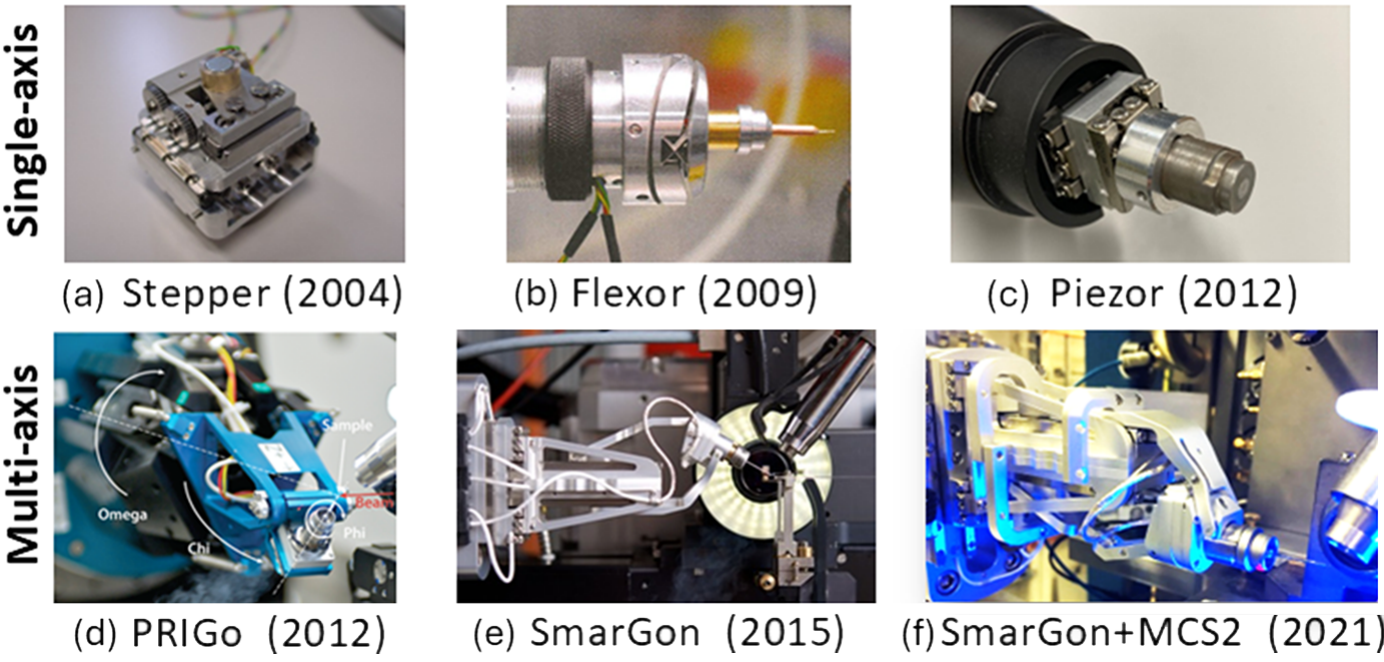
Evolution of the goniometer at the SLS. (*a*)–(*c*) Single-axis goniometers. (*d*)–(*f*) Multi-axis goniometers (Waltersperger *et al.*, 2015 ▸).

Several generations of single-axis goniometers were developed in the early days of SLS MX beamlines. We started with a compact design with a stack of two stepping motors (Pauluhn *et al.*, 2011 ▸) [Fig. 3 ▸(*a*)], which was replaced by a flexor device later (Fuchs *et al.*, 2014 ▸) [Fig. 3 ▸(*b*)]. When high-precision compact nano-positioning technology became available in 2008, we built a new goniometer using two piezo positioners (SmarAct GmbH) [Fig. 3 ▸(*c*)]. In parallel, we have been following the development of multi-axis goniometers at other synchrotron facilities, noticeably the mini-kappa design at the ESRF (Brockhauser *et al.*, 2013 ▸). Challenges were the precision required for collecting data from micrometre-sized crystals with micrometre-sized X-ray beams at synchrotron MX beamlines, avoiding collisions with beamline devices and self-shadowing on the detector. We developed the Parallel Robotics Inspired Goniometer (PRIGo), a new type of multi-axis goniometer with micrometre precision, large collision-free angular range and reduced self-shadowing (Waltersperger *et al.*, 2015 ▸) [Fig. 3 ▸(*d*)]. Based on a combination of serial and parallel kinematics, PRIGo utilized linear and rotary piezo positioners to emulate the movements of an arc. A calibration procedure was developed to reach the sphere of confusion <1 µm for Ω and <7 µm for χ, respectively. The PRIGo was installed at X06DA-PXIII in 2012.

To make the PRIGo technology accessible to beamlines at other facilities, we teamed up with the company SmarAct GmbH (Oldenburg, Germany) to create the next-generation device, SmarGon, in 2015 [Fig. 3 ▸(*e*)]. With enhanced precision and a simplified design for easier construction and calibration, SmarGon has since been deployed at beamlines at both the DLS and the SOLEIL (Source Optimisée de Lumière d’Énergie Intermédiaire du LURE) synchrotrons. Our collaboration with SmarAct continued to improve the system’s mechanical robustness, initialization procedures and calibration. We also developed a new control system (smargopolo) using the *Robot Operating System*. This latest generation, SmarGon-MCS2 [Fig. 3 ▸(*f*)], was successfully deployed at X06SA-PXI and X10SA-PXII in 2021 (Glettig *et al.*, 2024 ▸).

### 3.4. From MAD/SAD to Native-SAD phasing

The MAD and SAD techniques were revolutionary in MX phasing and became the main experimental phasing methods in the 2000s (Hendrickson, 2000 ▸; Hendrickson, 2014 ▸). The tunability of synchrotron radiation allowed easy access to the absorption edge of phasing elements to maximize both dispersive and anomalous signals. The success of cryogenic cooling made it possible to measure MAD from a single crystal, greatly improving the data accuracy required for the small amount of the anomalous signal. However, working with heavy elements that needed to be incorporated into protein was laborious and not consistently successful. Seleno­methio­nine derivatization later revolutionized *de novo* structure determination (Hendrickson *et al.*, 1990 ▸), significantly reducing the non-isomorphism problem and taking advantage of the convenience of using the 12.66 keV Se *K*-edge at synchrotrons. New methods, algorithms and powerful programs for data processing (Otwinowski & Minor, 1997 ▸; Leslie, 2006 ▸; Kabsch, 2010*b* ▸,*a*), phasing (de La Fortelle & Bricogne, 1997 ▸; Terwilliger & Berendzen, 1997 ▸; Schneider & Sheldrick, 2002 ▸; Sheldrick, 2010 ▸), density modification (Wang, 1985 ▸; Zhang & Main, 1990 ▸; Terwilliger, 2000 ▸; Sheldrick, 2002 ▸; Skubák & Pannu, 2011 ▸) and automatic model building (Perrakis, Morris & Lamzin, 1999 ▸; Cowtan, 2006 ▸; Terwilliger *et al.*, 2008 ▸; Pannu *et al.*, 2011 ▸; Usón & Sheldrick, 2018 ▸) gradually made the SAD phasing the first choice. As data accuracy continued to improve, another approach emerged: exploiting the anomalous signal from sulfur atoms in cysteine and me­thio­nine residues, which are natively present in most proteins, known as native-SAD (Hendrickson & Teeter, 1981 ▸; Liu *et al.*, 2012 ▸). However, since the anomalous sulfur signal is weak in the typical energy range of MX beamlines, it was necessary to increase the phasing signal-to-noise ratio. This involved reducing experimental systematic errors, using lower energy X-rays to enhance anomalous signals, or employing both strategies.

**Figure 4:**
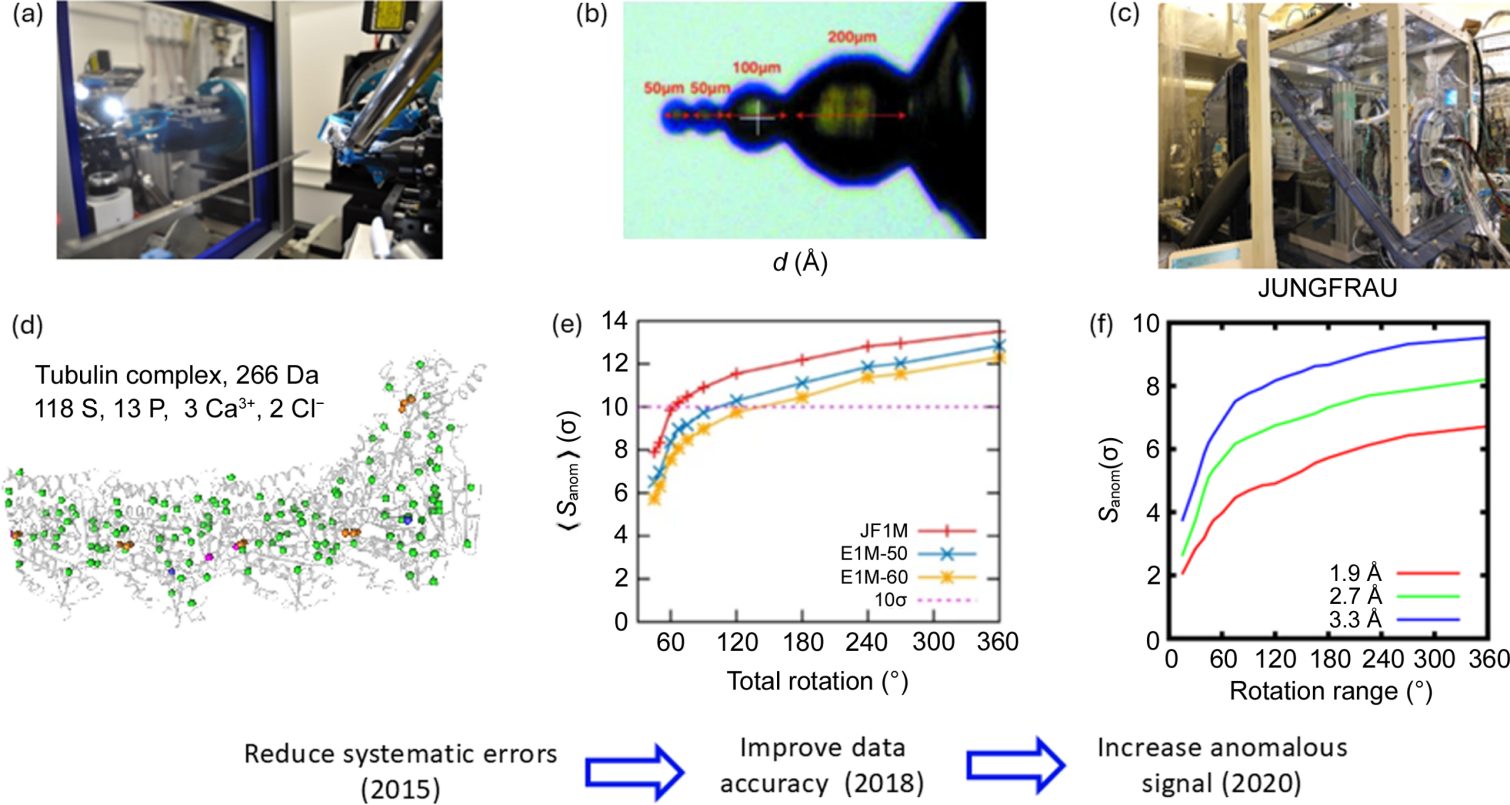
Instrumentation and methods development for native-SAD phasing. (*a*) Multi-axis goniometer (PRIGo) and PILATUS 2M detector at beamline X06DA-PXIII. (*b*) Deep-UV laser shaped crystal (Basu, Olieric *et al.*, 2019 ▸). (*c*) Helium sample environment at beamline BL-1A, PF, Japan. (*d*) The largest native-SAD structure [reproduced from Weinert *et al.* (2015 ▸)]. (*e*) Advantage of integrating detector for low-energy native-SAD [reproduced from Leonarski *et al.* (2018 ▸)]. (*f*) Harvesting anomalous signal at low-enegy with JUNGFRAU detector (unpublished data).

Various approaches have been developed to reduce measurement systematic errors. This included crystal alignment with a multi-axis goniometer to measure the Friedel pairs of reflections on the same diffraction image, ‘inverse beam’ strategy to collect Friedel pairs with similar X-ray dose, multi-orientation data collection with a three-circle goniometer for high true redundancy (Pal *et al.*, 2008 ▸) and high-redundancy data collection strategy to improve data precision (Liu, Chen *et al.*, 2011 ▸). In addition, Hendrickson demonstrated that multi-crystal averaging effectively reduces systematic errors and enhances signal-to-noise ratio (Liu, Zhang & Hendrickson, 2011 ▸) and applied it for *de novo* native-SAD structure determination (Liu *et al.*, 2012 ▸, 2013 ▸, 2014 ▸). We integrated these ideas by combining the use of the newly developed single-photon-counting detector (DECTRIS PILATUS) and the multi-axis goniometer (PRIGo). We proposed, using 6 keV energy, accessible at most tunable beamlines, a multi-orientation, low-dose, high-redundancy data collection strategy to effectively average out systematic errors by sampling crystal orientations, diffraction geometry and pixel-response variation of X-ray detector in one experiment (Weinert *et al.*, 2015 ▸) [Fig. 4 ▸(*a*)]. The method was used to solve the largest native-SAD structure and was used routinely at X06DA-PXIII (Basu, Finke *et al.*, 2019 ▸) [Fig. 4 ▸(*d*)].

Using X-rays close to the sulfur and phosphor absorption edges for anomalous scattering applications, including native-SAD phasing, has been pioneered by Stuhrmann and coworkers in the 1990s (Lehmann *et al.*, 1993 ▸; Stuhrmann *et al.*, 1995 ▸; Stuhrmann *et al.*, 1997 ▸). However, experimental complications from working at such low energies were not met at the time at MX beamlines. Dedicated beamlines were later constructed to reduce both air and sample absorption and to improve diffraction geometry and detector efficiency. Beamlines I23 at DLS (Wagner *et al.*, 2016 ▸) and BL-1A at the Photon Factory (PF) (Liebschner *et al.*, 2016 ▸) are two examples. They used either a vacuum or a helium sample environment to reduce air absorption, a kappa-goniometer to improve data completeness at low energy, and specialized detectors (one ‘cylindrical’ PILATUS 12M at I23 and two EIGER 4M in a V-shape configuration at BL-1A) with extra-low-energy calibrations. Both beamlines have been used successfully for native-SAD phasing. Still, at energies below 5 keV, sample absorption becomes significant, making it problematic to perform phasing routinely. Crystal shaping technology with a deep-UV laser developed at SPring-8 came to the rescue and was made available for routine use at BL-1A (Kitano *et al.*, 2005 ▸). We used spherically shaped crystals to demonstrate the advantages of low-energy native-SAD using 4.6 keV (2.7 Å) at BL-1A (Basu, Olieric *et al.*, 2019 ▸) [Figs. 4 ▸(*b*) and 4 ▸(*c*)].

Even with special calibration of single-photon-counting detectors, the ‘corner-effect’ at low energies remains problematic and limits the data accuracy required for native-SAD. We demonstrated that JUNGFRAU charge-integration eliminated the corner-effect and can produce more accurate data for low-energy phasing experiments (Leonarski *et al.*, 2018 ▸) [Fig. 4 ▸(*e*)]. In collaboration with BL-1A, we showed that enhanced anomalous signal at 3.75 keV (3.3 Å) could be harnessed effectively [Fig. 4 ▸(*f*)] by combining crystal-shaping, multi-orientation data collection and a JUNGFRAU 4M in a helium chamber [Fig. 4 ▸(*c*)]. Thanks to the kilohertz frame rate of the JUNGFRAU, 360° data sets could be collected with fine-phi slicing protocol at 100 ° s^−1^ fast rotation, which made the multi-orientation strategy fast and efficient. We applied this method to solve dozens *de novo* structures in 2020 (unpublished).

The significance of synchrotron experimental phasing can not be overstated (Hendrickson, 2023 ▸), but the success of experimental phasing challenged its own existence. With the advent of accurate protein structure prediction tools like *AlphaFold2* (Jumper *et al.*, 2021 ▸) and *RoseTTAFold* (Baek *et al.*, 2021 ▸), nearly all structures can now be solved by molecular replacement (Keegan *et al.*, 2024 ▸). Fortunately, instruments and methods developed at synchrotron beamlines go beyond experimental phasing. For example, multi-orientation allows improved coverage of reciprocal space for more complete and accurate data for MX structure refinement (Bricogne, 2020 ▸) and sampling of real space for X-ray tomography. The higher quality data with fewer systematic errors could help with studies on functional binding and drug design. In addition, it comes in handy when aligning chips and fixed targets for serial crystallography. The kilohertz data acquisition enables millisecond time-resolution in serial time-resolved crystallography (Leonarski, Nan *et al.*, 2023 ▸).

### 3.5. From *in situ* crystallography to multi-temperature MX

**Figure 5:**
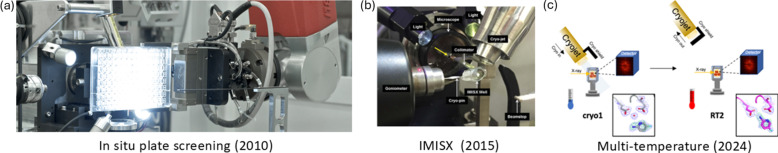
*In situ* crystallography: from RT to cryo and back. (*a*) *In situ* screening of a 96-well plate [reprinted with permission from Bingel-Erlenmeyer *et al.* (2011 ▸). Copyright (2011) American Chemical Society]. (*b*) *In meso in situ* serial crystallography (Huang *et al.*, 2016 ▸). (*c*) Apply IMISX chip for multi-temperature crystallography (Huang, Aumonier *et al.*, 2024 ▸).

Since the beginning of the 21st century, crystallography at cryogenic temperature has become the standard method at the synchrotron, which increased the X-ray dose limit to two orders of magnitude and greatly facilitated the logistics of transportation of fragile crystals (Garman & Schneider, 1997 ▸). Nevertheless, conducting initial diffraction screening directly in the crystallization container proved beneficial for further crystallization optimization and the evaluation of post-crystallization treatments (Martiel *et al.*, 2018 ▸). Jean-Luc Ferrer at the FIP beamline at the ESRF pioneered *in situ* diffraction from 96-well crystallization plates using a six-axis robot (Jacquamet *et al.*, 2004 ▸). We followed this idea and made an automated pipeline enabling the robotic transfer of crystallization plate from the storage hotel to the beamline for *in situ* diffraction screening at X06DA-PXIII in 2010 (Bingel-Erlenmeyer *et al.*, 2011 ▸). Users could change the beamline configuration to *in situ* diffraction from the GUI within 2 min and select crystallization plates from the Formulatrix Rock Imager 1000 or from a supplemental plate holder. A SCARA robot picked up the plate, transferred it to a Stäubli six-axis robot through the back window of the mini-hutch at X06DA-PXIII and the Stäubli presented the selected drop at the sample position for X-ray data collection [Fig. 5 ▸(*a*)]. This approach was later elaborated into a dedicated *in situ* diffraction screening beamline at the DLS (VMXi) reaching a much larger scale and higher automation (Sanchez-Weatherby *et al.*, 2019 ▸; Mikolajek *et al.*, 2023 ▸). To facilitate *in situ* data collection from smaller crystals, various miniaturized devices were developed with thin materials, including silicon films (Zarrine-Afsar *et al.*, 2012 ▸; Mueller *et al.*, 2015 ▸; Roedig *et al.*, 2016 ▸; Dunge *et al.*, 2024 ▸), silicon nitride windows (Coquelle *et al.*, 2015 ▸), polymers (Axford *et al.*, 2016 ▸; Baxter *et al.*, 2016 ▸; Schubert *et al.*, 2016 ▸; Guo *et al.*, 2018 ▸; Doak *et al.*, 2018 ▸; Cipriani *et al.*, 2012 ▸) and graphene (Sui *et al.*, 2016 ▸).

*In situ* crystallography for membrane protein crystals grown in lipidic cubic phases (LCP) in glass plates was another challenge due to the size of microcrystals and high diffraction background. The glass plate and viscosity of the LCP made crystal harvesting challenging. In collaboration with Caffrey, we developed a sandwich crystallization setup with two thin COC films, which allowed harvesting the whole LCP bolus containing microcrystals for *in situ* serial data collection (Huang *et al.*, 2015 ▸). The *in meso in situ* serial crystallography (IMISX) chips can be prepared with standard LCP crystallization robots. One advantage of the compact format of IMISX chip is that the whole chip can be cryo-cooled, allowing preparation of samples at users’ laboratories and sending them in a dryshipper (Dewar) for serial X-ray data collection at synchrotron beamlines (Huang *et al.*, 2016 ▸). The IMISX chip is compatible with most sample changers, enabling integration into automation workflows at MX beamlines [Fig. 5 ▸(*b*)], and the IMISX kit is commercially available via MiTeGen. Similar ideas have been pursued to improve throughput (Broecker *et al.*, 2018 ▸; Huang, Meier *et al.*, 2020 ▸) and automation (Felisaz *et al.*, 2019 ▸; Healey *et al.*, 2021 ▸).

Although the IMISX method was primarily developed for the determination of membrane protein structures (El Ghachi *et al.*, 2018 ▸; Apel *et al.*, 2019 ▸; Jaeger *et al.*, 2019 ▸; Olatunji *et al.*, 2021 ▸; Li *et al.*, 2021 ▸), it provided a general and adaptable platform for studying structures from cryogenic temperature to room temperature (RT) at standard MX beamlines (Huang, Olieric *et al.*, 2020 ▸). Easy access to multiple temperatures holds great potential for the study of dynamic processes (Douzou *et al.*, 1970 ▸; Horrell *et al.*, 2018 ▸; Yao *et al.*, 2021 ▸; Tsai *et al.*, 2022 ▸; Huang *et al.*, 2022 ▸; Greisman *et al.*, 2024 ▸; McLeod *et al.*, 2025 ▸). Recently, we have used it to reveal the changes in ligand binding of endothia­pepsin at multiple temperatures (Huang, Aumonier *et al.*, 2024 ▸) [Fig. 5 ▸(*c*)].

Another innovative approach to RT MX uses ultrasonic acoustic levitation to suspend liquid droplets containing protein crystals. The rapid spinning of the crystal within the levitating droplet enables efficient sampling of reciprocal space, while a fast frame-rate X-ray detector captures diffraction images in a manner similar to serial crystallography (Tsujino & Tomizaki, 2016 ▸). The method was later extended to levitate thin films as sample holders (Kepa *et al.*, 2022 ▸) and holds promise for studying dynamics through droplet mixing.

### 3.6. From micro-crystallography to serial crystallography

**Figure 6:**
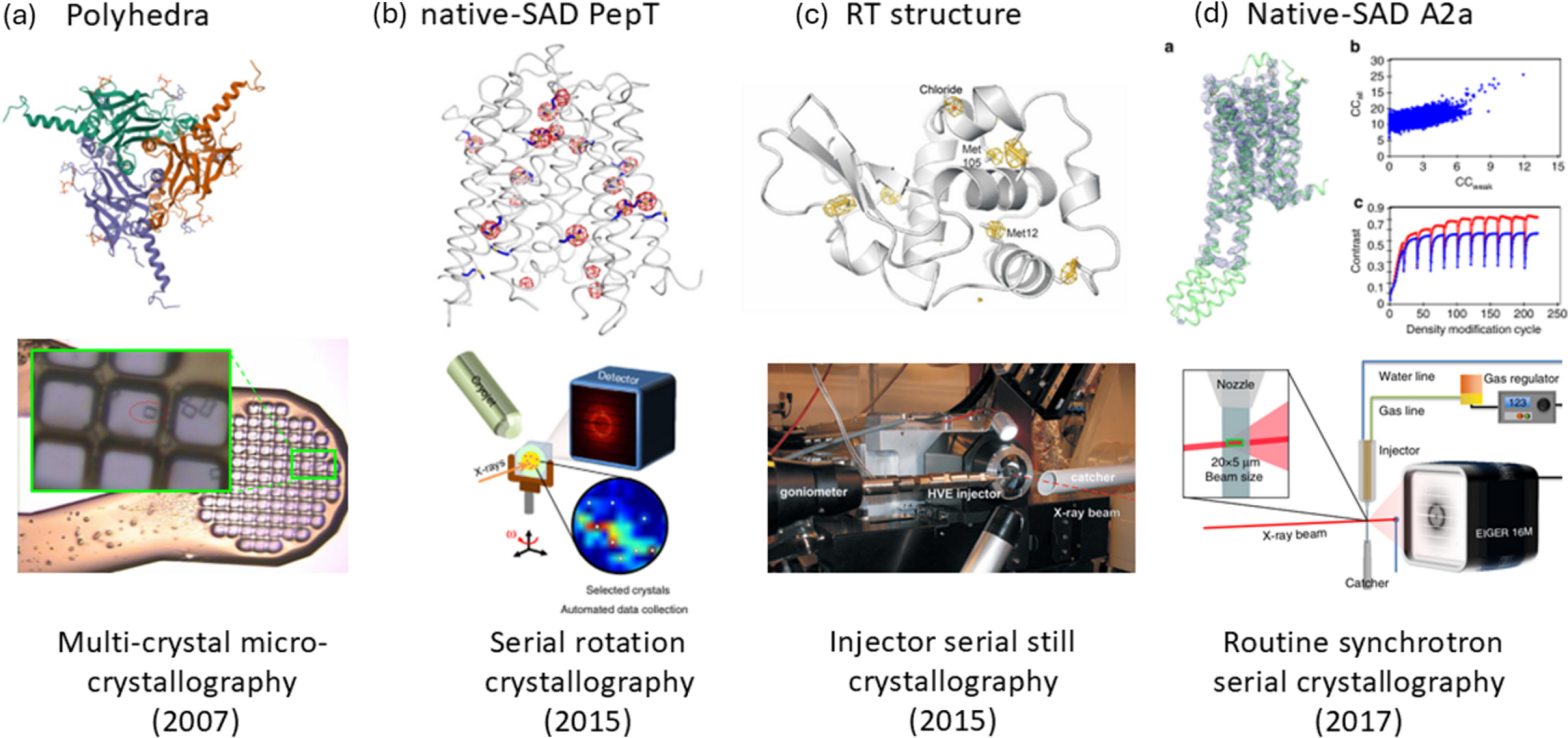
From micro-crystallography to serial crystallography. (*a*) The first polyhedra structure was solved by experimental phasing with multiple 5–10 micrometre-sized crystals (Coulibaly *et al.*, 2007 ▸). (*b*) High-quality data from serial rotation crystallography for native-SAD phasing of PepT (Huang *et al.*, 2018 ▸). (*c*) The first demonstration of RT structure determination with SFX-like serial still crystallography at a synchrotron [reproduced from Botha *et al.* (2015 ▸)]. (*d*) Demonstration of synchrotron serial crystallography in native-SAD phasing of A_2A_ (Weinert *et al.*, 2017 ▸).

Pioneered at the ESRF in the 1990s (Cusack *et al.*, 1998 ▸), protein micro-crystallography became instrumental in the structure determination of crystals <20 µm in size, such as those of G-protein coupled receptors (GPCRs) (Smith *et al.*, 2012 ▸). The micro-focusing capability was one unique feature at X06SA-PXI. The 5 µm focused beam allowed *de novo* structure determination of polyhedra from a few micrometre-sized crystals in 2007 (Coulibaly *et al.*, 2007 ▸) [Fig. 6 ▸(*a*)] and of microcrystalline insulin (Wagner *et al.*, 2009 ▸). The high flux density reduced the crystal lifetime to a few seconds due to radiation damage (Owen *et al.*, 2006 ▸; Holton, 2009 ▸). Therefore, multi-crystal merging was routinely used to obtain a complete data set (Coulibaly *et al.*, 2009 ▸). Soon after, the micro-beam feature was introduced at X10SA-PXII to meet the industry’s demand for membrane protein drug discovery projects.

It quickly became critical that automation was necessary to identify and center micro-crystals. This led to the development of fast grid scans, which became indispensable in locating micro-crystals grown in LCP, as LCP turns opaque upon cooling. The diffraction-based grid scan was first reported at the Stanford Synchrotron Radiation Lightsource (SSRL) in 2007 (Song *et al.*, 2007 ▸). Similar implementations with small beams were realized at DLS (Aishima *et al.*, 2010 ▸), ESRF (Bowler *et al.*, 2010 ▸) and APS (Cherezov *et al.*, 2009 ▸). Using an EIGER detector, we achieved 100 Hz grid scan with real-time diffraction hit analysis using a combination of continuous 2D scan, DISTL spot finding (Zhang *et al.*, 2006 ▸) and our *DA+* software suite that uses messaging and streaming technologies (Wojdyla *et al.*, 2016 ▸). We also explored X-ray imaging-based crystal identification methods, namely scanning transmission X-ray microscope and full-field X-ray imaging (Martiel, Huang *et al.*, 2020 ▸). Alternative methods based on UV fluorescence (Stepanov *et al.*, 2011 ▸) and SONNIC were developed (Calero *et al.*, 2014 ▸; Madden *et al.*, 2013 ▸). These methods could locate micrometre-sized crystals with zero or near-zero X-ray doses, but they did not provide information on X-ray diffraction quality and required additional instruments. Therefore, we focused on the X-ray diffraction-based method and automated serial rotation crystallography by collecting a small wedge of data (typically 10°) from each crystal (*CY+*) (Basu, Kaminski *et al.*, 2019 ▸), similar to *MeshAndCollect* at ESRF (Zander *et al.*, 2015 ▸) or the *ZOO* method at SPring-8 (Hirata *et al.*, 2019 ▸). We further developed an automated data processing and merging pipeline to process each data wedge separately, select isomorphous data sets, and merge them until the desired data quality was reached (Basu, Kaminski *et al.*, 2019 ▸). The *CY+* GUI and automation in serial data processing made serial rotation crystallography more accessible to our user community. A simple and deterministic data-scaling and selection method was later developed with Diederichs, particularly effective for experimental phasing by anomalous diffraction (Assmann *et al.*, 2020 ▸). Unlike conventional crystallography, which uses one single crystal, serial crystallography consumes more samples. Still, averaging can minimize systematic experimental errors effectively and produce highly accurate data for the most challenging experimental phasing experiment, native-SAD (Huang *et al.*, 2018 ▸) [Fig. 6 ▸(*b*)]. The same is true for detecting weak binding ligands (Pearce *et al.*, 2017 ▸) and extracting excited states from time-resolved crystallography data (Ursby & Bourgeois, 1997 ▸; Genick, 2007 ▸).

Following the success of SFX at XFEL facilities in the 2010s (Boutet *et al.*, 2019 ▸), synchrotron facilities embraced the new technology (Stellato *et al.*, 2014 ▸; Henkel & Oberthür, 2024 ▸; Gati *et al.*, 2014 ▸). They incorporated innovations in serial sample delivery (Sierra *et al.*, 2018 ▸), the measurement of still diffraction images, and novel data processing and merging techniques (White *et al.*, 2012 ▸; Sauter *et al.*, 2014 ▸). In collaboration with our beamline partner, the Max Planck Institute, we demonstrated SSX using serial sample delivery with the high-viscosity extrusion injector, micro-focused X-ray beam and high frame-rate detector PILATUS 6M at beamline X10SA-PXII (Botha *et al.*, 2015 ▸). We showed that high-quality RT data can be obtained and alluded to the possibility of studying protein structural dynamics using SSX [Fig. 6 ▸(*c*)]. A similar demonstration was conducted with a CCD detector at ESRF (Nogly *et al.*, 2015 ▸). Later, we applied the method to several proteins and made the method routine with a faster EIGER detector at beamline X06SA-PXI (Weinert *et al.*, 2017 ▸) [Fig. 6 ▸(*d*)]. The method was further enhanced by utilizing wide-bandpass or polychromatic (‘pink’) beams to improve data quality and reduce sample consumption (Meents *et al.*, 2017 ▸; Martin-Garcia *et al.*, 2019 ▸; Tolstikova *et al.*, 2019 ▸).

### 3.7. Time-resolved serial crystallography at SwissFEL and SLS

**Figure 7:**
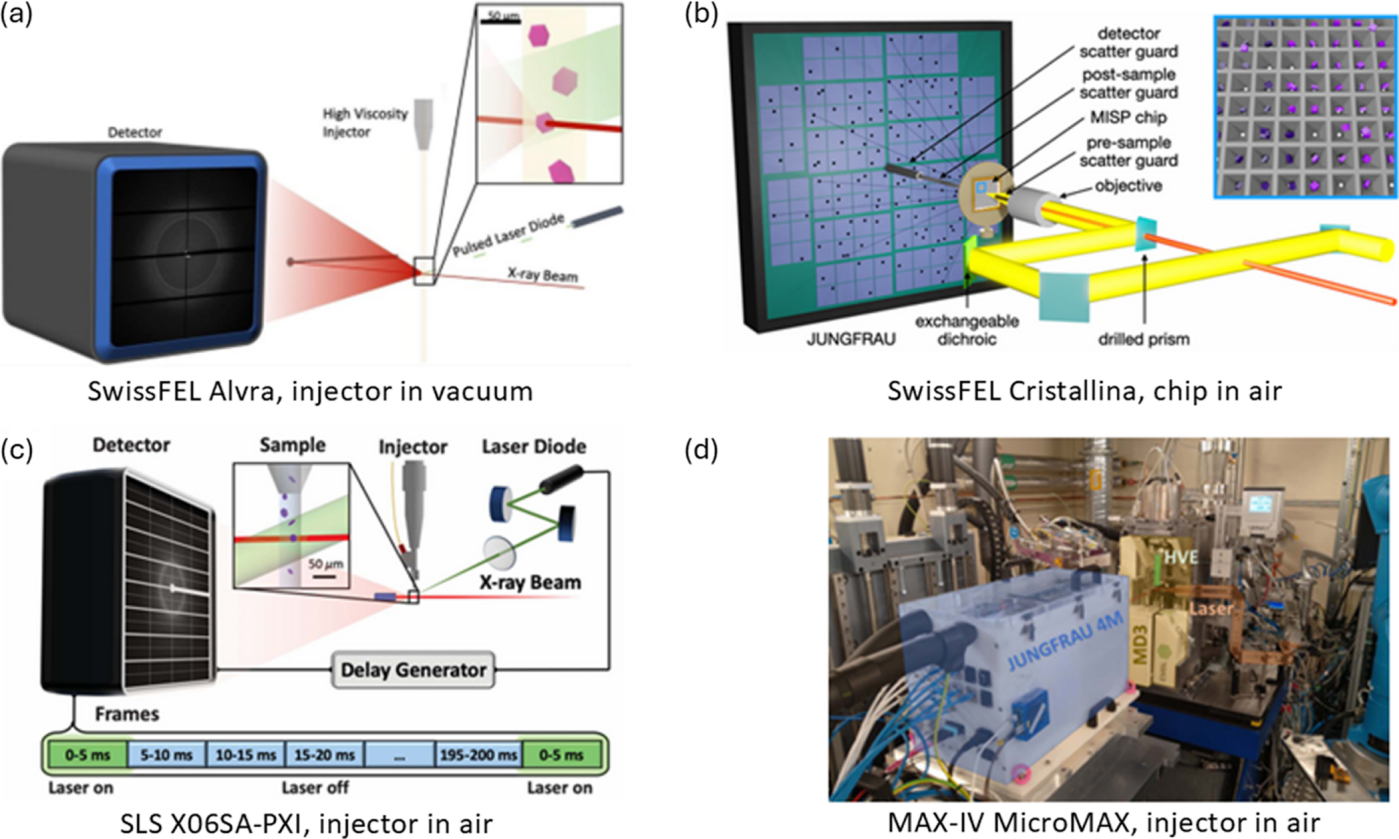
Time-resolved crystallography at (*a*) SwissFEL Alvra [reproduced from Mous *et al.* (2022 ▸), reprinted with permission from AAAS], (*b*) SwissFEL Cristallina (Gotthard, Flores-Ibarra *et al.*, 2024 ▸), (*c*) SLS X06SA-PXI [reproduced from Weinert *et al.* (2019 ▸), reprinted with permission from AAAS] and (*d*) MAX-IV MicroMAX (Leonarski, Nan *et al.*, 2023 ▸).

SFX revived time-resolved MX and pushed time-resolution to femtoseconds (Moffat & Lattman, 2023 ▸). The first SwissFEL experimental station Alvra offered SFX with injector-based sample delivery methods and a JUNGFRAU 16M detector (Milne *et al.*, 2017 ▸) [Fig. 7 ▸(*a*)]. Automated experiment logging and data processing improved the efficiency and feedback of SFX. Alvra attracted internal and external expert user groups and established itself as a productive SFX facility. Highlights include the dynamics and mechanism of a light-driven sodium pump (Skopintsev *et al.*, 2020 ▸) and a chloride pump (Mous *et al.*, 2022 ▸), the drug release from tubulin (Wranik *et al.*, 2023 ▸), DNA repair process (Maestre-Reyna *et al.*, 2023 ▸; Christou *et al.*, 2023 ▸), and the first molecular events of vision (Gruhl *et al.*, 2023 ▸). At the Bernina experimental station, a dedicated instrument (SwissMX), including the robotic sample changer TELL (Martiel, Buntschu *et al.*, 2020 ▸), was developed to provide SFX with fixed-target sample delivery (Ingold *et al.*, 2019 ▸). The SwissMX was further developed at the Cristallina experimental station soon after, aiming to increase user experiment capacity with automated fixed-target approaches [Fig. 7 ▸(*b*)]. The first fixed-target pump–probe experiment has been published recently (Gotthard, Flores-Ibarra *et al.*, 2024 ▸).

In collaboration with Standfuss’s group at PSI, the synergy between SwissFEL and SLS was effectively leveraged to develop time-resolved serial synchrotron crystallography (TR-SSX) at X06SA-PXI. The millisecond time resolution enabled us to capture large conformational changes during the pumping cycle of bacteriorhodopsin (Weinert *et al.*, 2019 ▸) [Fig. 7 ▸(*c*)] and the reaction of a blue light photoreceptor domain (Gotthard, Mous *et al.*, 2024 ▸). The TR-SSX data also complemented the SFX data in the studies of a light-driven chloride pump (Mous *et al.*, 2022 ▸) and drug release mechanism from tubulin (Wranik *et al.*, 2023 ▸).

The development of the *Jungfraujoch* kilohertz data-acquisition system (Leonarski, Brückner *et al.*, 2023 ▸) with the JUNGFRAU detector also enabled probing of multiple time points from microseconds to seconds in one experiment sequence at synchrotron sources. In collaboration with the MicroMAX team at the first fourth-generation synchrotron, MAX-IV, we showed that a static serial data set could be obtained within minutes and that a 1 ms resolution was achieved (Leonarski, Nan *et al.*, 2023 ▸) [Fig. 7 ▸(*d*)]. In addition, JUNGFRAU can be operated in burst mode to reach microsecond-level time resolution (Sikorski *et al.*, 2023 ▸).

### 3.8. MX beamline automation, high-throughput screening and unattended beamline operation

Beamline automation as a global effort had a profound impact on modern synchrotron crystallography (Arzt *et al.*, 2005 ▸; Soltis *et al.*, 2008 ▸). MX beamline operations have evolved from manual sample mounting to robotic sample exchange, from on-site data collection to remote operation, from human oversight to fully unattended operation.

The cryogenic crystallography workflow made robotic sample exchange routinely attainable, leading to the development of sample changers at most synchrotrons over the last two decades (Muchmore *et al.*, 2000 ▸; Cohen *et al.*, 2002 ▸; Ohana *et al.*, 2004 ▸; Cork *et al.*, 2006 ▸; Ueno *et al.*, 2004 ▸; Arzt *et al.*, 2005 ▸; Cipriani *et al.*, 2006 ▸; Papp *et al.*, 2017 ▸; O’Hea *et al.*, 2018 ▸). We started with the Cryogenic Automated Transfer System (CATS) system (Ohana *et al.*, 2004 ▸) for its versatility. Indeed, the system offered wet-mounting, dry-mounting and *in situ* plate screening capabilities (Jacquamet *et al.*, 2004 ▸). Later, inspired by the DLS BART system (O’Hea *et al.*, 2018 ▸), we developed high-throughput enabling large-capacity sample loader (TELL) for SwissFEL and SLS with a large capacity dewar holding 480 samples in UniPuck format (Martiel, Buntschu *et al.*, 2020 ▸). The SUNA gripper developed at Deutsches Elektronen-Synchrotron (DESY) was later replaced with our gripper based on the original ALS design (Cork *et al.*, 2006 ▸). This in-house development improved reliability, speed and compatibility for both cryogenic and RT sample exchange.

Thanks to automation, the remote operation of our MX beamlines steadily increased until the start of the COVID-19 pandemic in 2020. The SLS MX beamlines never stopped operation throughout the pandemic and offered beam time to academics and industry with dedicated fast access for drug discovery programs against COVID-19 (Shin *et al.*, 2020 ▸; Gao, Qin *et al.*, 2021 ▸; Gao, Zhu *et al.*, 2021 ▸; Qin *et al.*, 2023 ▸; Sutanto *et al.*, 2021 ▸; Huang, Metz *et al.*, 2024 ▸).

The sophistication of automation and continuous improvement of fast X-ray detectors gradually increased the throughput so that hundreds of data sets could be collected daily. This throughput gain was transformative and made crystallographic fragment-based screening a reality (Davies & Tickle, 2012 ▸). The XChem team at the DLS pioneered this work with streamlined processes, from sample preparation to data management and analysis. They built a full X-ray screening facility at beamline I04-1 in 2015 (Fearon *et al.*, 2024 ▸). Similar facilities were constructed at other synchrotrons (Lima *et al.*, 2020 ▸; Wollenhaupt *et al.*, 2021 ▸; Cornaciu *et al.*, 2021 ▸; Barthel *et al.*, 2024 ▸; Huang *et al.*, 2025 ▸). The SLS fast fragment and compound screening pipeline (FFCS) was built in 2020, focusing on industrial applications (Kaminski *et al.*, 2022 ▸; Stegmann *et al.*, 2023 ▸). Our service includes the complete pipeline from crystallization, soaking, crystal harvesting to X-ray data collection and analysis.

With the increasing throughput, unattended beamline operation became a highly valued mode of operation by users. A fully automated pipeline from crystal mounting to structure refinement was pioneered for Eli Lilly and Company at LRL-CAT, APS (Wasserman *et al.*, 2012 ▸). MASSIF-1 was developed to automate characterization and data collection at the ESRF, taking crystal size, flux and X-ray dose into account (Bowler *et al.*, 2015 ▸). At SPring-8, the automatic data collection was optimized for micro-crystallography (Hirata *et al.*, 2019 ▸). Our implementation focused on higher throughput for industrial applications. The combination of the fast TELL sample changer, rapid grid scan and short data collection allowed us to process 25 samples per hour before the SLS 2.0 upgrade (Smith *et al.*, 2023 ▸). Current development focuses on further improving throughput, making it available for RT data collection and extending the automation to SSX applications.

### 3.9. Impact beyond MX

Advancements in beamline instrumentation and automation have the potential to influence fields beyond MX. For example, small-angle X-ray scattering tensor tomography (SAS-TT) is a powerful technique for studying the multiscale architecture of hierarchical structures (Liebi *et al.*, 2015 ▸). SAS-TT measurements typically require a few hundred 2D SAS scans at various sample orientations, which is often a manual and lengthy process. To make SAS-TT accessible to a broader user base, we automated the complete SAS-TT tomogram sampling and reduced data acquisition time by leveraging the precision of the SmarGon multi-axis goniometer and the speed of 2D scanning at the X06SA-PXI beamline (Appel *et al.*, 2024 ▸). An experiment that used to take up to four days was realized in 1.2 h. In addition, the cryogenic MX setup allows automatic sample exchange and reduces radiation damage for SAS-TT applications in life science. The automation expertise and productivity enhancements developed in MX could be extended to other synchrotron beamlines, including tomography (Albers *et al.*, 2024 ▸), coherent diffraction imaging and spectroscopy.

## 4. Future development and SLS 2.0 upgrade

In the last decade, we were excited to see revolutionary extensions of the structural biology toolbox, from MX, SAXS and NMR to cryoEM (Kühlbrandt, 2014 ▸), cryoET (Turk & Baumeister, 2020 ▸), microED (Nannenga *et al.*, 2014 ▸), X-ray bio-imaging (Albers *et al.*, 2024 ▸) and accurate structure prediction (Jumper *et al.*, 2021 ▸), allowing comprehensive multi-scale investigation of a wide range of biological samples. Except for NMR, other experimental techniques primarily capture static structures at cryogenic temperature. As for the atomic resolution technique, 190000 X-ray static structures (https://biosync.rcsb.org) have been determined since the first protein diffraction at SSRL 50 years ago (Phillips *et al.*, 1976 ▸) thanks to bright synchrotron lights, routine experimental phasing, fast X-ray detectors, and automation in laboratories and beamlines. The cryogenic MX will continue to routinely provide high-resolution structures with even higher throughput.

High-resolution cryogenic structures are the cornerstone for investigating the functions of biomolecules. However, this static perspective can overlook the subtle yet critical conformational changes and dynamic movements that proteins undergo during catalysis, ligand binding and allosteric regulation. That also, to some extent, limited modeling and molecular dynamics simulation due to the lack of high-resolution structural data at physiological temperatures and along energy landscapes. Because *AlphaFold2/3* was trained predominantly on cryogenic temperatures, it also faces limitations in capturing the dynamic molecular interactions vital for many biological processes and identifying potential therapeutic compounds (Agarwal & McShan, 2024 ▸). Therefore, it is essential to determine 3D structures at biologically relevant temperatures and time scales to gain a deeper understanding of protein function and dynamics (Henzler-Wildman & Kern, 2007 ▸; van den Bedem & Fraser, 2015 ▸; Nam & Wolf-Watz, 2023 ▸). Integration of ‘dynamic’ experimental structures, AI-based structure prediction, modeling and molecular dynamics simulation will make protein design and structure-based drug discovery more accurate, efficient and accessible (Reardon, 2024 ▸).

After mastering cryogenic MX at third-generation synchrotrons, one next grand challenge is whether fourth-generation synchrotrons and XFEL facilities can offer cryocrystallography-like user-friendliness and throughput to emerging dynamic MX applications. Impressive development has already been accomplished (Henkel & Oberthür, 2024 ▸); dedicated on-site sample preparation laboratories were built at and near synchrotron and XFEL facilities (Han *et al.*, 2021 ▸), and specialized crystal transportation and crystallization setups were developed for RT samples (Baxter *et al.*, 2016 ▸). Such RT sample preparation facilities and sample transportation methods are certainly needed. Still, we would reason that the true potential of synchrotron and XFEL RT MX will be severely limited if the sample preparation and X-ray data collection are coupled in time and space. Then, the success of beam time will be heavily dependent on the timing of delicate and often not very reproducible crystal growth and the capacity of sample laboratories at synchrotron facilities. We foresee active developments to improve the productivity of dynamic MX applications at synchrotron and XFEL facilities in the coming decade. This calls for corresponding development in data analysis tools as well. Finally, we encourage the user community to explore MX beyond static cryogenic structures. Our recent developments in the context of the SLS 2.0 upgrade are briefly described below.

### 4.1. From cryogenic temperature back to RT

Despite the recent revival of RT MX and its appealing applications in protein dynamics and drug discovery (Fischer, 2021 ▸; Thorne, 2023 ▸), sample logistics severely limit its broad adoption. Indeed, transporting delicate crystals at RT from the research laboratories to the synchrotron facilities is challenging, and the synchronization between sample preparation and beam time presents another obstacle. In contrast, the sample logistics problem has been solved in cryo-crystallography, which enabled users worldwide to optimize their samples, harvest them and preserve them in optimal conditions ahead of MX measurements. These samples are stored and shipped to the facility when beam time becomes available. This cryogenic workflow has provided a continuous supply of samples to the facilities, enabling the exponential growth of experimental structures determined at synchrotron facilities (https://biosync.rcsb.org/). To address the logistics issues at RT, we recently proposed a *Cryo2RT* workflow to determine high-quality RT structures from previously cryocooled crystals (Huang, Aumonier *et al.*, 2024 ▸). This seemingly unconventional method leverages the advantages of conventional cryogenic MX, allowing us to circumvent the RT sample logistics problems by incorporating the proven cryogenic sample preparation, transportation and beamline automation workflow. Since nearly all crystals can be cryo-cooled, the reverse process could also be applicable in general (Kriminski *et al.*, 2002 ▸; Juers & Matthews, 2001 ▸; Juers *et al.*, 2007 ▸). The method can be readily adapted to standard MX beamlines with a humidity control device, *e.g.* HC-Lab (https://www.arinax.com) and Watershed (https://www.mitegen.com).

Conventional cryogenic crystallography has distinct advantages, including high resolution and sensitivity to weak binders, but cryo-cooling often drives the system to one low-temperature state. Recent studies show that catalytic efficiency is related to sampling multiple conformational states, challenging the traditional static view of enzyme catalysis (Yabukarski *et al.*, 2022 ▸). The temperature-dependent binding modes and conformations could be accessible with RT crystallography (Fraser *et al.*, 2009 ▸; Weik & Colletier, 2010 ▸; Fraser *et al.*, 2011 ▸; Fischer *et al.*, 2015 ▸; Keedy *et al.*, 2018 ▸; Greisman *et al.*, 2024 ▸), but the RT experiment has other challenges. The foremost is reproducibility – RT structures are affected by the crystallization conditions, the crystal harvesting conditions and the potential structure changes during X-ray data collection, such as dehydration, rehydration, X-ray beam heating, radiation damage *etc*. The experimental conditions must be carefully controlled and varied systematically to capture relevant structure ensembles.

Radiation damage is another challenge. The femtosecond pulses provided by XFEL sources enabled high-resolution structure determination without radiation damage by the ‘diffraction before destruction’ principle in SFX. The SFX damage-free structures serve as the ‘ground truth’ (Hirata *et al.*, 2014 ▸; Williams *et al.*, 2025 ▸) and are particularly important in studying metalloproteins (Kern *et al.*, 2015 ▸; Bowman *et al.*, 2016 ▸; Hirata *et al.*, 2014 ▸; Suga *et al.*, 2015 ▸). At synchrotrons, however, a balance between reaching high resolution and minimizing X-ray dose needs to be found. For example, multi-crystal and serial crystallography could increase attainable diffraction resolution from small crystals by distributing X-ray dose (de la Mora *et al.*, 2020 ▸). Unlike X-ray damage at 100 K, radiation damage at RT is both dose and dose-rate dependent (Southworth-Davies *et al.*, 2007 ▸; Rajendran *et al.*, 2011 ▸; Owen *et al.*, 2012 ▸; Warkentin *et al.*, 2012 ▸, 2011 ▸). The radiation damage is also temperature and time dependent, and the damage mechanism is less understood (Warkentin *et al.*, 2013 ▸). We have been using fast frame-rate X-ray detectors to track radiation damage in millisecond timescales at RT (Rajendran *et al.*, 2011 ▸; Huang *et al.*, 2015 ▸). The recent advances in kilohertz MX with JUNGFRAU detector (Tolstikova *et al.*, 2019 ▸; Leonarski, Nan *et al.*, 2023 ▸; Leonarski, Brückner *et al.*, 2023 ▸) could even ‘outrun’ slow radiation damage processes (Warkentin *et al.*, 2013 ▸; Thorne, 2023 ▸). With increased flux at higher energy in fourth-generation synchrotrons and X-ray detectors with high-*Z* sensors, high-energy MX has the potential to further reduce radiation damage (Dickerson & Garman, 2019 ▸) and improve diffraction resolution (Jaho *et al.*, 2024 ▸). Note that the ‘low reproducibility’ of RT MX experiments could complicate the situation, calling for more systematic and comprehensive studies on dynamic radiation damage processes and their impact on RT structures.

Finally, analysis and interpretation of RT structures are not part of the standard MX practices (Woldeyes *et al.*, 2014 ▸). The corresponding data processing, refinement (Burnley *et al.*, 2012 ▸; Du *et al.*, 2023 ▸), modeling (Riley *et al.*, 2021 ▸) and visualization pipelines need to be developed and deployed at future MX beamlines.

### 4.2. New dimensions in macromolecular crystallography

After 100 years of structural biology research, static 3D structures of proteins can be determined to high resolution and predicted with high accuracy. The next forefront is dynamic structural biology to understand the intricate behaviors of proteins beyond static views, uncover how molecular conformations shift in response to environmental factors (*e.g.* temperature, pH), binding events or catalytic cycles in a time-resolved manner. Temperature, binding pose and time are readily accessible in MX. A temperature range from glass transition to physiological temperatures (∼200–300 K) can be controlled with standard cryojets, which were previously used primarily to cool down crystals to 100 K. Various small format fixed-targets developed for SSX can be used directly for multi-temperature MX data collection. This large temperature window enables us to probe the thermodynamics and kinetics of protein dynamics, changes in water structures and ligand interactions at atomic resolution (Rasmussen *et al.*, 1992 ▸; Tilton *et al.*, 1992 ▸; Horrell *et al.*, 2018 ▸; Ringe & Petsko, 2003 ▸; Greisman *et al.*, 2024 ▸). For example, a 10–20°C temperature change can alter ligand binding (Huang *et al.*, 2022 ▸; Du *et al.*, 2023 ▸). This small temperature change is within the reach of X-ray induced beam heating (Kriminski *et al.*, 2003 ▸; Warren *et al.*, 2019 ▸; Baxter *et al.*, 2024 ▸), raising concerns and calling for careful experimental control from X-ray beam characteristics (size, profile, flux) to sample preparations.

Temperature-dependent and time-resolved MX will enable us to study dynamic structural enzymology (Douzou *et al.*, 1970 ▸; Tilton *et al.*, 1992 ▸; Fraser *et al.*, 2009 ▸; Bhabha *et al.*, 2015 ▸; Beyerlein *et al.*, 2017 ▸; Kupitz *et al.*, 2017 ▸; Keedy *et al.*, 2018 ▸; Bradford *et al.*, 2021 ▸; Yao *et al.*, 2021 ▸; Stachowski *et al.*, 2022 ▸; Greisman *et al.*, 2024 ▸; McLeod *et al.*, 2025 ▸; Banari *et al.*, 2025 ▸). By controlling the diffusion process and altering the reaction rate at different temperatures, we could capture reaction mechanisms and dynamics of conformational changes (Tsai *et al.*, 2022 ▸). Advanced temperature-jump techniques using infrared lasers could probe relaxation processes from micro- to millisecond resolution utilizing fast-frame-rate X-ray detectors (Wolff *et al.*, 2023 ▸). Alternatively, fast laser heating combined with re-vitrification can trap dynamics in microsecond resolutions, as shown in time-resolved cryo-electron microscopy (Lorenz, 2024 ▸). This innovative microsecond cryo-trapping techniques could be adapted to MX at synchrotron beamlines.

Novel instrumentations have been explored to control binding events by mixing substrate/ligand and protein crystals. Mix-and-extrude with microchannels and micronozzles (Vakili *et al.*, 2023 ▸), liquid application method with fixed-target (Mehrabi *et al.*, 2023 ▸), acoustic-based drop-on-drop (Fuller *et al.*, 2017 ▸) and levitation methods (Tsujino & Tomizaki, 2016 ▸; Kepa *et al.*, 2022 ▸) have been developed to control the initiation of the reaction within the crystals and capture intermediate states that occur as the enzyme catalyzes its reaction in millisecond to second time scales. However, most methods require specialized instruments and significant expertise. To advance this area of research, these techniques must be democratized and made available to a broader range of researchers. Achieving this involves developing standardized protocols, user-friendly software and automated systems that can handle the complexity of these experiments on standard MX beamlines.

### 4.3. MX opportunities at the SLS 2.0 upgrade and SwissFEL

**Figure 8:**
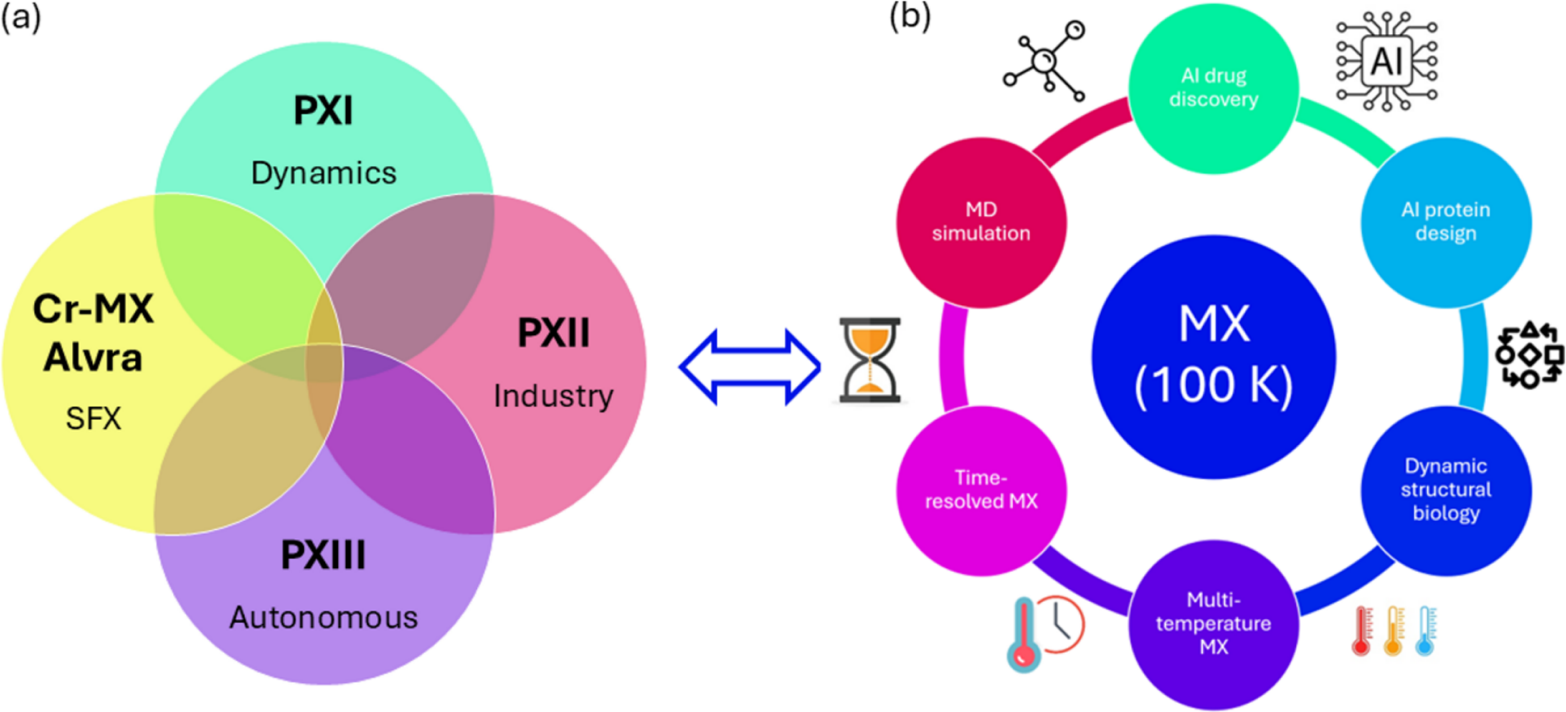
SLS 2.0 upgrade and MX applications. (*a*) Synergies among three MX beamlines at SLS 2.0 and two end-stations at SwissFEL. (*b*) MX applications.

The SLS 2.0 upgrade is currently underway (Willmott & Braun, 2024 ▸). We have redesigned our three MX beamlines to exploit the brighter source and address MX’s future needs (Fig. 8 ▸). We strive to increase throughput for conventional cryogenic MX further, make RT MX routine and explore new dimensions in MX. Fully unattended beamline operation will be offered to academic and industry users, while hands-on, on-site support will be provided to explore new opportunities. There will be a paradigm shift in the usage of beam time. In the past, a significant part of beam time was dedicated to solving new structures of large molecular complexes and membrane proteins by screening weakly diffracting crystals and their derivatives. These demands have been moderated by the recent breakthroughs in cryoEM (Kühlbrandt, 2014 ▸) and *AlphaFold2* (Jumper *et al.*, 2021 ▸). In the future, more beam time will be available to study structures and their dynamics.

**Figure 9:**
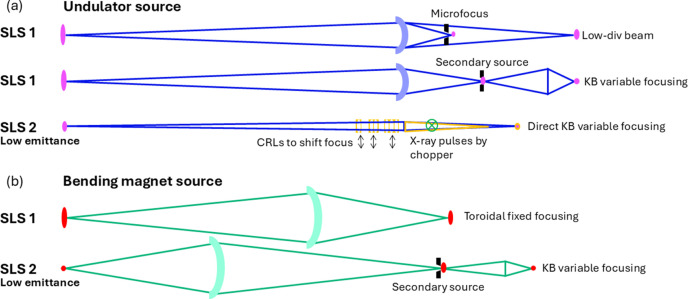
Evolution of X-ray optics design of MX beamlines from SLS to SLS 2.0. (*a*) The low emittance source favors direct focusing at undulator sources. (*b*) Small source size enables variable focusing with acceptable beam divergence at the bending magnet source. The illustrations are from top views.

The higher machine energy (2.7 GeV), low source emittance of 157 × 10 pm rad, and advances in undulator technology, monochromator, and X-ray mirrors and refractive optics make it easier to expand X-ray characteristics for MX beamlines. The X-ray optics design has evolved accordingly (Fig. 9 ▸). At the SLS, previous X06SA-PXI and X10SA-PXII started with direct focusing, and the beam-defining apertures near the sample position were used to make micro-beams at the cost of flux. Later, both beamlines were changed to two-stage focusing for more flexible beam size and divergence control [Fig. 9 ▸(*a*)]. With the low emittance source of the SLS 2.0, two-stage focusing is no longer necessary for undulator beamlines X06SA-PXI and X10SA-PXII. A secondary optics element could serve to tune the primary focusing. For a bending magnet source, where the large acceptance angle in the horizontal plane and beam collimating in the vertical plane are required, two-stage optics is advantageous. Thanks to the much-reduced source size at the SLS 2.0, moderate focusing is sufficient to achieve a micro-beam with less beam aberration and maintain an acceptable beam divergence for the new X06DA-PXIII [Fig. 9 ▸(*b*)].

The new X06SA-PXI and X10SA-PXII beamlines will deliver micro-focused low-divergence X-rays with a monochromatic flux >10^13^ photons s^−1^ at 12.4 keV. The beam size can be varied from 1 to 100 µm with KB mirrors to match the crystal size. In addition, we will use two sets of 2D beryllium compound refractive lenses for quick beam resizing for industry applications at X10SA-PXII. The new bending magnet X06DA-PXIII beamline combines a toroidal prefocusing/collimating mirror and a KB-focusing mirror system via a horizontal secondary source to achieve a variable beam size from 15 to 100 µm with flux of 10^12^ photons s^−1^ at 12.4 keV. The X-ray bandwidth and energy range will be extended to 1% at PXI, 30 keV at PXI and PXII, and 4 keV at PXIII. The main beamline characteristics are listed in Table 1 ▸. All three new beamlines can perform conventional MX, each with its own specific focus: PXI for dynamics and X-ray imaging, PXII for industry applications, PXIII for autonomous operation [Fig. 8 ▸(*a*)].

We are developing a new MX software suite to accelerate and advance MX research at the new beamlines at the SLS 2.0. The suite integrates beamline device control; orchestrates MX experiments; accelerates data acquisition; assists structure determination and interpretation; facilitates information flow for experimental feedback; and interfaces to beamline developers, operations and users. We aim to achieve ‘intelligent’ beamline operation with real-time data analysis, machine-learning-based experimental feedback and steering to enable autonomous experiments for routine and advanced MX applications.

Increases in source brightness, advances in detector technology and sample delivery methods, and expansion in MX experimental techniques also lead to a formidable data challenge. For example, kilohertz data acquisition will be routine for rotation and serial crystallography to enhance throughput and capture dynamic processes. Innovative and sustainable IT solutions are needed to release the full potential of future light sources. We will address such challenges by exploiting edge computing, utilizing high-performance computing (HPC) infrastructure at PSI, and exploring cloud-based services. Recently, we have enabled kilohertz data analysis in *Jungfraujoch* so that initial image analysis, compression and reduction could be carried out for each image before data are written to storage. With ever-powerful GPU and FPGA technologies and available AI/ML data analysis methods, data-driven experimental feedback and steering could be implemented in the future. The pre-processed data will feed local HPC or cloud service for complete analysis and optimization. We expect disruptive innovative data analysis methods will be developed for future experiments.

Three MX beamlines at the SLS 2.0 and two endstations at SwissFEL will offer our user community a comprehensive set of high-resolution structure techniques, from high-throughput applications at cryogenic and around RT to damage-free and time-resolved structures [Fig. 8 ▸(*b*)]. The enriched experimental structures could advance physics-based molecular dynamics simulations and AI-driven structure prediction methods in structural biology. This will expand our knowledge of protein structure and function and ultimately accelerate *de novo* protein design, discoveries in drug development and beyond. After the 10000 static structures at the SLS, we are at a new beginning with SLS 2.0 and SwissFEL.

## Supplementary Material

Supporting table and figures. DOI: 10.1107/S1600577525005016/sze5006sup1.pdf

## Figures and Tables

**Table 1:** Main characteristics of three MX beamlines at the SLS and SLS 2.0

	X06SA-PXI		X10SA-PXII		X06DA-PXIII	
	SLS	SLS 2.0	SLS	SLS 2.0	SLS	SLS 2.0
Start year	2001	2025	2004	2025	2008	2025
Machine energy (GeV)	2.4	2.7	2.4	2.7	2.4	2.7
Source	U19, 1.9 m	U17, 3 m	U19, 1.9 m	U17, 3 m	SB, 2.7 T	2.1 T
Photon source size, divergence (µm, µrad in FWHM at 12.4 keV)	202 × 23 135 × 25	47 × 10 33 × 28	202 × 23 135 × 25	47 × 10 33 × 28	130 × 20 402 × 308	16 × 16 263 × 251
Energy (keV)	5.7–17.5	5–30	6–20	5–30	5–17.5	4–15
Energy resolution	10^−4^	10^−4^, 10^−2^	10^−4^	10^−4^	10^−4^	10^−4^
Flux (photons s^−1^ at 12.4 keV)	10^12^	10^13^, 10^15^	10^12^	10^13^	10^11^	10^12^
Beam size (µm in FWHM)	5–100	1–100	15–100	1–100	90 × 45 (h × v)	15–100
Goniometer	SmarGon	SmarGon	SmarGon	SmarGon	PRIGo	SmarGon
Detector	EIGER 16M	EIGER 16M, JUNGFRAU 9M	EIGER2 16M	EIGER2 16M	PILATUS 2M	PILATUS4 2M
Sample changer	TELL	TELL2	TELL	TELL2	TELL	TELL2

## References

[bb1] Abramson, J., Smirnova, I., Kasho, V., Verner, G., Kaback, H. R. & Iwata, S. (2003). *Science* **301**, 610–615. 10.1126/science.1088196 12893935

[bb2] Agarwal, V. & McShan, A. C. (2024). *Nat. Chem. Biol.* **20**, 950–959. 10.1038/s41589-024-01638-w PMC11956457 38907110

[bb3] Aishima, J., Owen, R. L., Axford, D., Shepherd, E., Winter, G., Levik, K., Gibbons, P., Ashton, A. & Evans, G. (2010). *Acta Cryst.* D**66**, 1032–1035. 10.1107/S0907444910028192 PMC6691516 20823554

[bb4] Albers, J., Nikolova, M., Svetlove, A., Darif, N., Lawson, M. J., Schneider, T. R., Schwab, Y., Bourenkov, G. & Duke, E. (2024). *J. Synchrotron Rad.* **31**, 186–194. 10.1107/S160057752300944X PMC10833423 37971957

[bb5] Apel, A., Cheng, R. K. Y., Tautermann, C. S., Brauchle, M., Huang, C., Pautsch, A., Hennig, M., Nar, H. & Schnapp, G. (2019). *Structure* **27**, 427–438.e5. 10.1016/j.str.2018.10.027 30581043

[bb6] Appel, C., Schmeltz, M., Rodriguez-Fernandez, I., Anschuetz, L., Nielsen, L. C., Panepucci, E., Marijolovic, T., Wakonig, K., Ivanovic, A., Bonnin, A., Leonarski, F., Wojdyla, J., Tomizaki, T., Guizar-Sicairos, M., Smith, K., Beale, J. H., Glettig, W., McAuley, K., Bunk, O., Wang, M. & Liebi, M. (2024). *arXiv*:2406.13238.

[bb7] Arzt, S., Beteva, A., Cipriani, F., Delageniere, S., Felisaz, F., Förstner, G., Gordon, E., Launer, L., Lavault, B., Leonard, G., Mairs, T., McCarthy, A., McCarthy, J., McSweeney, S., Meyer, J., Mitchell, E., Monaco, S., Nurizzo, D., Ravelli, R., Rey, V., Shepard, W., Spruce, D., Svensson, O. & Theveneau, P. (2005). *Prog. Biophys. Mol. Biol.* **89**, 124–152. 10.1016/j.pbiomolbio.2004.09.003 15910915

[bb8] Assmann, G. M., Wang, M. & Diederichs, K. (2020). *Acta Cryst.* D**76**, 636–652. 10.1107/S2059798320006348 PMC7336379 32627737

[bb9] Axford, D., Aller, P., Sanchez-Weatherby, J. & Sandy, J. (2016). *Acta Cryst.* F**72**, 313–319. 10.1107/S2053230X16004386 PMC4822989 27050266

[bb10] Baek, M., DiMaio, F., Anishchenko, I., Dauparas, J., Ovchinnikov, S., Lee, G. R., Wang, J., Cong, Q., Kinch, L. N., Schaeffer, R. D., Millán, C., Park, H., Adams, C., Glassman, C. R., DeGiovanni, A., Pereira, J. H., Rodrigues, A. V., van Dijk, A. A., Ebrecht, A. C., Opperman, D. J., Sagmeister, T., Buhlheller, C., Pavkov-Keller, T., Rathinaswamy, M. K., Dalwadi, U., Yip, C. K., Burke, J. E., Garcia, K. C., Grishin, N. V., Adams, P. D., Read, R. J. & Baker, D. (2021). *Science* **373**, 871–876. 10.1126/science.abj8754 PMC7612213 34282049

[bb11] Banari, A., Samanta, A. K., Munke, A., Laugks, T., Bajt, S., Grünewald, K., Marlovits, T. C., Küpper, J., Maia, F. R. N. C., Chapman, H. N., Oberthür, D. & Seuring, C. (2025). *Nat. Methods*https://doi.org/10.1038/s41592-025-02659-6. 10.1038/s41592-025-02659-6 40312512

[bb12] Barthel, T., Benz, L., Basler, Y., Crosskey, T., Dillmann, A., Förster, R., Fröling, P., Dieguez, C. G., Gless, C., Hauß, T., Hellmig, M., Jänisch, L., James, D., Lennartz, F., Mijatovic, J., Oelker, M., Scanlan, J. W., Weber, G., Wollenhaupt, J., Mueller, U., Dobbek, H., Wahl, M. C. & Weiss, M. S. (2024). *Appl. Res.* **3**, e202400110.

[bb13] Basu, S., Finke, A., Vera, L., Wang, M. & Olieric, V. (2019). *Acta Cryst.* D**75**, 262–271. 10.1107/S2059798319003103 PMC6450063 30950397

[bb14] Basu, S., Kaminski, J. W., Panepucci, E., Huang, C.-Y., Warshamanage, R., Wang, M. & Wojdyla, J. A. (2019). *J. Synchrotron Rad.* **26**, 244–252. 10.1107/S1600577518016570 PMC6337882 30655492

[bb15] Basu, S., Olieric, V., Leonarski, F., Matsugaki, N., Kawano, Y., Takashi, T., Huang, C.-Y., Yamada, Y., Vera, L., Olieric, N., Basquin, J., Wojdyla, J. A., Bunk, O., Diederichs, K., Yamamoto, M. & Wang, M. (2019). *IUCrJ* **6**, 373–386. 10.1107/S2052252519002756 PMC6503925 31098019

[bb16] Baxter, E. L., Aguila, L., Alonso-Mori, R., Barnes, C. O., Bonagura, C. A., Brehmer, W., Brunger, A. T., Calero, G., Caradoc-Davies, T. T., Chatterjee, R., Degrado, W. F., Fraser, J. M., Ibrahim, M., Kern, J., Kobilka, B. K., Kruse, A. C., Larsson, K. M., Lemke, H. T., Lyubimov, A. Y., Manglik, A., McPhillips, S. E., Norgren, E., Pang, S. S., Soltis, S. M., Song, J., Thomaston, J., Tsai, Y., Weis, W. I., Woldeyes, R. A., Yachandra, V., Yano, J., Zouni, A. & Cohen, A. E. (2016). *Acta Cryst.* D**72**, 2–11. 10.1107/S2059798315020847 PMC4756618 26894529

[bb17] Baxter, J. M., Hutchison, C. D. M., Fadini, A., Maghlaoui, K., Cordon-Preciado, V., Morgan, R. M. L., Agthe, M., Horrell, S., Tellkamp, F., Mehrabi, P., Pfeifer, Y., Müller-Werkmeister, H. M., von Stetten, D., Pearson, A. R. & van Thor, J. J. (2024). *J. Am. Chem. Soc.* **146**, 16394–16403. 10.1021/jacs.3c12883 PMC11191680 38848551

[bb18] Beyerlein, K. R., Dierksmeyer, D., Mariani, V., Kuhn, M., Sarrou, I., Ottaviano, A., Awel, S., Knoska, J., Fuglerud, S., Jönsson, O., Stern, S., Wiedorn, M. O., Yefanov, O., Adriano, L., Bean, R., Burkhardt, A., Fischer, P., Heymann, M., Horke, D. A., Jungnickel, K. E. J., Kovaleva, E., Lorbeer, O., Metz, M., Meyer, J., Morgan, A., Pande, K., Panneerselvam, S., Seuring, C., Tolstikova, A., Lieske, J., Aplin, S., Roessle, M., White, T. A., Chapman, H. N., Meents, A. & Oberthuer, D. (2017). *IUCrJ* **4**, 769–777. 10.1107/S2052252517013124 PMC5668862 29123679

[bb19] Bhabha, G., Biel, J. T. & Fraser, J. S. (2015). *Acc. Chem. Res.* **48**, 423–430. 10.1021/ar5003158 PMC4334266 25539415

[bb20] Bingel-Erlenmeyer, R., Olieric, V., Grimshaw, J. P. A., Gabadinho, J., Wang, X., Ebner, S. G., Isenegger, A., Schneider, R., Schneider, J., Glettig, W., Pradervand, C., Panepucci, E. H., Tomizaki, T., Wang, M. & Schulze-Briese, C. (2011). *Cryst. Growth Des.* **11**, 916–923.

[bb21] Böge, M. (2002). *Proceedings of the 8th European Particle Accelerator Conference (EPAC2002)*, 3–7 June 2002, Paris, France, pp. 39–43.

[bb22] Botha, S., Nass, K., Barends, T. R. M., Kabsch, W., Latz, B., Dworkowski, F., Foucar, L., Panepucci, E., Wang, M., Shoeman, R. L., Schlichting, I. & Doak, R. B. (2015). *Acta Cryst.* D**71**, 387–397. 10.1107/S1399004714026327 25664750

[bb23] Boutet, S., Fromme, P. & Hunter, M. S. (2019). *X-ray free electron lasers: A revolution in structural biology*. Cham: Springer Nature.

[bb24] Bowler, M. W., Guijarro, M., Petitdemange, S., Baker, I., Svensson, O., Burghammer, M., Mueller-Dieckmann, C., Gordon, E. J., Flot, D., McSweeney, S. M. & Leonard, G. A. (2010). *Acta Cryst.* D**66**, 855–864. 10.1107/S0907444910019591 20693684

[bb25] Bowler, M. W., Nurizzo, D., Barrett, R., Beteva, A., Bodin, M., Caserotto, H., Delagenière, S., Dobias, F., Flot, D., Giraud, T., Guichard, N., Guijarro, M., Lentini, M., Leonard, G. A., McSweeney, S., Oskarsson, M., Schmidt, W., Snigirev, A., von Stetten, D., Surr, J., Svensson, O., Theveneau, P. & Mueller-Dieckmann, C. (2015). *J. Synchrotron Rad.* **22**, 1540–1547. 10.1107/S1600577515016604 PMC4629869 26524320

[bb26] Bowman, S. E. J., Bridwell Rabb, J. & Drennan, C. L. (2016). *Acc. Chem. Res.* **49**, 695–702. 10.1021/acs.accounts.5b00538 PMC4838947 26975689

[bb27] Bradford, S. Y. C., El Khoury, L., Ge, Y., Osato, M., Mobley, D. L. & Fischer, M. (2021). *Chem. Sci.* **12**, 11275–11293. 10.1039/d1sc02751d PMC8447925 34667539

[bb28] Bricogne, G. (2020). *Structural Biology in Drug Discovery*, pp. 211–251. Wiley.

[bb29] Brockhauser, S., Ravelli, R. B. G. & McCarthy, A. A. (2013). *Acta Cryst.* D**69**, 1241–1251. 10.1107/S0907444913003880 PMC3689527 23793150

[bb30] Broecker, J., Morizumi, T., Ou, W.-L., Klingel, V., Kuo, A., Kissick, D. J., Ishchenko, A., Lee, M.-Y., Xu, S., Makarov, O., Cherezov, V., Ogata, C. M. & Ernst, O. P. (2018). *Nat. Protoc.* **13**, 260–292. 10.1038/nprot.2017.135 29300389

[bb31] Burnley, B. T., Afonine, P. V., Adams, P. D. & Gros, P. (2012). *Elife* **1**, e00311. 10.7554/eLife.00311 PMC3524795 23251785

[bb32] Calero, G., Cohen, A. E., Luft, J. R., Newman, J. & Snell, E. H. (2014). *Acta Cryst.* F**70**, 993–1008. 10.1107/S2053230X14016574 PMC4118793 25084371

[bb33] Casanas, A., Warshamanage, R., Finke, A. D., Panepucci, E., Olieric, V., Nöll, A., Tampé, R., Brandstetter, S., Förster, A., Mueller, M., Schulze-Briese, C., Bunk, O. & Wang, M. (2016). *Acta Cryst.* D**72**, 1036–1048. 10.1107/S2059798316012304 PMC5013597 27599736

[bb34] Chapman, H. N. (2018). *Nat. Methods* **15**, 774–775. 10.1038/s41592-018-0150-8 30275571

[bb35] Cherezov, V., Hanson, M. A., Griffith, M. T., Hilgart, M. C., Sanishvili, R., Nagarajan, V., Stepanov, S., Fischetti, R. F., Kuhn, P. & Stevens, R. C. (2009). *J. R. Soc. Interface* **6**, S587–S597. 10.1098/rsif.2009.0142.focus PMC2843980 19535414

[bb36] Christou, N.-E., Apostolopoulou, V., Melo, D. V. M., Ruppert, M., Fadini, A., Henkel, A., Sprenger, J., Oberthuer, D., Günther, S., Pateras, A., Rahmani Mashhour, A., Yefanov, O. M., Galchenkova, M., Reinke, P. Y. A., Kremling, V., Scheer, T. E. S., Lange, E. R., Middendorf, P., Schubert, R., De Zitter, E., Lumbao-Conradson, K., Herrmann, J., Rahighi, S., Kunavar, A., Beale, E. V., Beale, J. H., Cirelli, C., Johnson, P. J. M., Dworkowski, F., Ozerov, D., Bertrand, Q., Wranik, M., Bacellar, C., Bajt, S., Wakatsuki, S., Sellberg, J. A., Huse, N., Turk, D., Chapman, H. N. & Lane, T. J. (2023). *Science* **382**, 1015–1020. 10.1126/science.adj4270 38033070

[bb37] Cipriani, F., Felisaz, F., Launer, L., Aksoy, J.-S., Caserotto, H., Cusack, S., Dallery, M., di-Chiaro, F., Guijarro, M., Huet, J., Larsen, S., Lentini, M., McCarthy, J., McSweeney, S., Ravelli, R., Renier, M., Taffut, C., Thompson, A., Leonard, G. A. & Walsh, M. A. (2006). *Acta Cryst.* D**62**, 1251–1259. 10.1107/S0907444906030587 17001102

[bb38] Cipriani, F., Röwer, M., Landret, C., Zander, U., Felisaz, F. & Márquez, J. A. (2012). *Acta Cryst.* D**68**, 1393–1399. 10.1107/S0907444912031459 22993093

[bb39] Cohen, A. E., Ellis, P. J., Miller, M. D., Deacon, A. M. & Phizackerley, R. P. (2002). *J. Appl. Cryst.* **35**, 720–726. 10.1107/s0021889802016709 PMC4041710 24899734

[bb40] Coquelle, N., Brewster, A. S., Kapp, U., Shilova, A., Weinhausen, B., Burghammer, M. & Colletier, J.-P. (2015). *Acta Cryst.* D**71**, 1184–1196. 10.1107/S1399004715004514 PMC4427202 25945583

[bb41] Cork, C., O’Neill, J., Taylor, J. & Earnest, T. (2006). *Acta Cryst.* D**62**, 852–858. 10.1107/S0907444906014120 16855300

[bb42] Cornaciu, I., Bourgeas, R., Hoffmann, G., Dupeux, F., Humm, A.-S., Mariaule, V., Pica, A., Clavel, D., Seroul, G., Murphy, P. & Márquez, J. A. (2021). *JoVE* **172**, e62491. 10.3791/62491 34152315

[bb43] Coulibaly, F., Chiu, E., Gutmann, S., Rajendran, C., Haebel, P. W., Ikeda, K., Mori, H., Ward, V. K., Schulze-Briese, C. & Metcalf, P. (2009). *Proc. Natl Acad. Sci. USA* **106**, 22205–22210. 10.1073/pnas.0910686106 PMC2799703 20007786

[bb44] Coulibaly, F., Chiu, E., Ikeda, K., Gutmann, S., Haebel, P. W., Schulze-Briese, C., Mori, H. & Metcalf, P. (2007). *Nature* **446**, 97–101. 10.1038/nature05628 17330045

[bb45] Cowtan, K. (2006). *Acta Cryst.* D**62**, 1002–1011. 10.1107/S0907444906022116 16929101

[bb46] Cusack, S., Belrhali, H., Bram, A., Burghammer, M., Perrakis, A. & Riekel, C. (1998). *Nat. Struct. Biol.* **5**, 634–637. 10.1038/1325 9699611

[bb47] Dauter, Z. (1999). *Acta Cryst.* D**55**, 1703–1717. 10.1107/s0907444999008367 10531520

[bb48] Davies, T. G. & Tickle, I. J. (2012). *Top. Curr. Chem.* **317**, 33–59. 10.1007/128_2011_179 21678136

[bb49] de La Fortelle, E. & Bricogne, G. (1997). *Methods Enzymol.* **276**, 472–494. 10.1016/S0076-6879(97)76073-7 27799110

[bb50] de la Mora, E., Coquelle, N., Bury, C. S., Rosenthal, M., Holton, J. M., Carmichael, I., Garman, E. F., Burghammer, M., Colletier, J.-P. & Weik, M. (2020). *Proc. Natl Acad. Sci. USA* **117**, 4142–4151. 10.1073/pnas.1821522117 PMC7049125 32047034

[bb51] Dickerson, J. L. & Garman, E. F. (2019). *J. Synchrotron Rad.* **26**, 922–930. 10.1107/S160057751900612X 31274414

[bb52] Diederichs, K. & Wang, M. (2017). *Methods Mol. Biol.* **1607**, 239–272. 10.1007/978-1-4939-7000-1_10 28573576

[bb53] Diez, J., Wang, M., Pohl, E., Tomizaki, T., Bertrand, A., Chen, Q., Dietrich, P., Ingold, G., Knecht, M., Meents, A., Olieric, V., Panepucci, E., Pauluhn, A., Pradervand, C., Roccamante, M., Schneider, R., Walthert, I., Zimoch, E. & Schulze-Briese, C. (2007). *Synchrotron Radiat. News* **20**(4), 19–22.

[bb54] Dinapoli, R., Bergamaschi, A., Henrich, B., Horisberger, R., Johnson, I., Mozzanica, A., Schmid, E., Schmitt, B., Schreiber, A., Shi, X. & Theidel, G. (2011). *Nucl. Instrum. Methods Phys. Res. A* **650**, 79–83.

[bb55] Doak, R. B., Nass Kovacs, G., Gorel, A., Foucar, L., Barends, T. R. M., Grünbein, M. L., Hilpert, M., Kloos, M., Roome, C. M., Shoeman, R. L., Stricker, M., Tono, K., You, D., Ueda, K., Sherrell, D. A., Owen, R. L. & Schlichting, I. (2018). *Acta Cryst.* D**74**, 1000–1007. 10.1107/S2059798318011634 PMC6173051 30289410

[bb56] Douzou, P., Sireix, R. & Travers, F. (1970). *Proc. Natl Acad. Sci. USA* **66**, 787–792. 10.1073/pnas.66.3.787 PMC283119 5269241

[bb57] Du, S., Wankowicz, S. A., Yabukarski, F., Doukov, T., Herschlag, D. & Fraser, J. S. (2023). *Methods Enzymol.* **688**, 223–254. 10.1016/bs.mie.2023.06.009 PMC10637719 37748828

[bb58] Dunge, A., Phan, C., Uwangue, O., Bjelcic, M., Gunnarsson, J., Wehlander, G., Käck, H. & Brändén, G. (2024). *IUCrJ* **11**, 831–842. 10.1107/S2052252524006134 PMC11364032 39072424

[bb59] Eikenberry, E. F., Brönnimann, C., Hülsen, G., Toyokawa, H., Horisberger, R., Schmitt, B., Schulze-Briese, C. & Tomizaki, T. (2003). *Nucl. Instrum. Methods Phys. Res. A* **501**, 260–266.

[bb60] El Ghachi, M., Howe, N., Huang, C.-Y., Olieric, V., Warshamanage, R., Touzé, T., Weichert, D., Stansfeld, P. J., Wang, M., Kerff, F. & Caffrey, M. (2018). *Nat. Commun.* **9**, 1078. 10.1038/s41467-018-03477-5 PMC5852022 29540682

[bb61] Engel, C., Gubbey, T., Neyer, S., Sainsbury, S., Oberthuer, C., Baejen, C., Bernecky, C. & Cramer, P. (2017). *Cell* **169**, 120–131.e22. 10.1016/j.cell.2017.03.003 28340337

[bb62] Fearon, D., Powell, A., Douangamath, A., Dias, A., Tomlinson, C. W. E., Balcomb, B. H., Aschenbrenner, J. C., Aimon, A., Barker, I. A., Bertram, F., Brandão–Neto, J., Coe, P. A., Collins, P., Dunnett, L. E., Fairhead, M., Gildea, R. J., Golding, M., Gorrie–Stone, T., Hathaway, P. V., Koekemoer, L., Krojer, T., Lithgo, R. M., Maclean, E. M., Marples, P. G., Mikolajek, H., Ni, X., Nidamarthi, K. H. V., O’Donnell, G., Skyner, R., Talon, R., Thompson, W., Watt, G., Wild, C. F., Williams, M. A., Winokan, M., Wright, N. D., Winter, G., Shotton, E. J. & von Delft, F. (2024). *Appl. Res.* **4**, e202400192.

[bb63] Felisaz, F., Sinoir, J., Papp, G., Pica, A., Bowler, M. W., Murphy, P., Hoffmann, G., Zander, U., Lopez-Marrero, M., Janocha, R., Giraud, T., Svensson, O., Popov, S., Leonard, G., Mueller-Dieckmann, C., Márquez, J. A., McCarthy, A. A. & Cipriani, F. (2019). *AIP Conf. Proc.* **2054**, 050009.

[bb64] Ferretti, S., Hamon, J., de Kanter, R., Scheufler, C., Andraos-Rey, R., Barbe, S., Bechter, E., Blank, J., Bordas, V., Dammassa, E., Decker, A., Di Nanni, N., Dourdoigne, M., Gavioli, E., Hattenberger, M., Heuser, A., Hemmerlin, C., Hinrichs, J., Kerr, G., Laborde, L., Jaco, I., Núñez, E. J., Martus, H.-J., Quadt, C., Reschke, M., Romanet, V., Schaeffer, F., Schoepfer, J., Schrapp, M., Strang, R., Voshol, H., Wartmann, M., Welly, S., Zécri, F., Hofmann, F., Möbitz, H. & Cortés-Cros, M. (2024). *Nature* **629**, 443–449. 10.1038/s41586-024-07350-y PMC11078746 38658754

[bb65] Fischer, M. (2021). *Q. Rev. Biophys.* **54**, e1. 10.1017/S0033583520000128 33413726

[bb66] Fischer, M., Shoichet, B. K. & Fraser, J. S. (2015). *Chembiochem* **16**, 1560–1564. 10.1002/cbic.201500196 PMC4539595 26032594

[bb67] Förster, A., Brandstetter, S. & Schulze-Briese, C. (2019). *Philos. Trans. A Math. Phys. Eng. Sci.* **377**, 20180241. 10.1098/rsta.2018.0241 PMC6501887 31030653

[bb68] Fraser, J. S., Clarkson, M. W., Degnan, S. C., Erion, R., Kern, D. & Alber, T. (2009). *Nature* **462**, 669–673. 10.1038/nature08615 PMC2805857 19956261

[bb69] Fraser, J. S., van den Bedem, H., Samelson, A. J., Lang, P. T., Holton, J. M., Echols, N. & Alber, T. (2011). *Proc. Natl Acad. Sci. USA* **108**, 16247–16252. 10.1073/pnas.1111325108 PMC3182744 21918110

[bb70] Fuchs, M. R., Pradervand, C., Thominet, V., Schneider, R., Panepucci, E., Grunder, M., Gabadinho, J., Dworkowski, F. S. N., Tomizaki, T., Schneider, J., Mayer, A., Curtin, A., Olieric, V., Frommherz, U., Kotrle, G., Welte, J., Wang, X., Maag, S., Schulze-Briese, C. & Wang, M. (2014). *J. Synchrotron Rad.* **21**, 340–351. 10.1107/S160057751400006X PMC3945418 24562555

[bb71] Fuller, F. D., Gul, S., Chatterjee, R., Burgie, E. S., Young, I. D., Lebrette, H., Srinivas, V., Brewster, A. S., Michels-Clark, T., Clinger, J. A., Andi, B., Ibrahim, M., Pastor, E., de Lichtenberg, C., Hussein, R., Pollock, C. J., Zhang, M., Stan, C. A., Kroll, T., Fransson, T., Weninger, C., Kubin, M., Aller, P., Lassalle, L., Bräuer, P., Miller, M. D., Amin, M., Koroidov, S., Roessler, C. G., Allaire, M., Sierra, R. G., Docker, P. T., Glownia, J. M., Nelson, S., Koglin, J. E., Zhu, D., Chollet, M., Song, S., Lemke, H., Liang, M., Sokaras, D., Alonso-Mori, R., Zouni, A., Messinger, J., Bergmann, U., Boal, A. K., Bollinger, J. M. Jr, Krebs, C., Högbom, M., Phillips, G. N. Jr, Vierstra, R. D., Sauter, N. K., Orville, A. M., Kern, J., Yachandra, V. K. & Yano, J. (2017). *Nat. Methods* **14**, 443–449. 10.1038/nmeth.4195 PMC5376230 28250468

[bb72] Gao, X., Qin, B., Chen, P., Zhu, K., Hou, P., Wojdyla, J. A., Wang, M. & Cui, S. (2021). *Acta Pharm Sin. B* **11**, 237–245. 10.1016/j.apsb.2020.08.014 PMC7467110 32895623

[bb73] Gao, X., Zhu, K., Qin, B., Olieric, V., Wang, M. & Cui, S. (2021). *Nat. Commun.* **12**, 2843. 10.1038/s41467-021-23118-8 PMC8121815 33990585

[bb74] Garman, E. F. & Schneider, T. R. (1997). *J. Appl. Cryst.* **30**, 211–237.

[bb75] Gasparotto, P., Barba, L., Stadler, H.-C., Assmann, G., Mendonça, H., Ashton, A. W., Janousch, M., Leonarski, F. & Béjar, B. (2024). *J. Appl. Cryst.* **57**, 931–944. 10.1107/S1600576724003182 PMC11299607 39108821

[bb76] Gati, C., Bourenkov, G., Klinge, M., Rehders, D., Stellato, F., Oberthür, D., Yefanov, O., Sommer, B. P., Mogk, S., Duszenko, M., Betzel, C., Schneider, T. R., Chapman, H. N. & Redecke, L. (2014). *IUCrJ* **1**, 87–94. 10.1107/S2052252513033939 PMC4062088 25075324

[bb77] Genick, U. K. (2007). *Acta Cryst.* D**63**, 1029–1041. 10.1107/S0907444907038164 17881820

[bb78] Glettig, W., Buntschu, D., Omelcenko, A., Panepucci, E. & Wang, M. (2024). *Proceedings of the 12th International Conference on Mechanical Engineering Design of Synchrotron Radiation Equipment and Instrumentation (MEDSI2023)*, 6–10 November 2023, Beijing, China. THOAM02.

[bb79] Gotthard, G., Flores-Ibarra, A., Carrillo, M., Kepa, M. W., Mason, T. J., Stegmann, D. P., Olasz, B., Pachota, M., Dworkowski, F., Ozerov, D., Pedrini, B. F., Padeste, C., Beale, J. H. & Nogly, P. (2024). *IUCrJ* **11**, 749–761. 10.1107/S2052252524005591 PMC11364036 38980142

[bb80] Gotthard, G., Mous, S., Weinert, T., Maia, R. N. A., James, D., Dworkowski, F., Gashi, D., Furrer, A., Ozerov, D., Panepucci, E., Wang, M., Schertler, G. F. X., Heberle, J., Standfuss, J. & Nogly, P. (2024). *IUCrJ* **11**, 792–808. 10.1107/S2052252524005608 PMC11364019 39037420

[bb81] Goverde, C. A., Pacesa, M., Goldbach, N., Dornfeld, L. J., Balbi, P. E. M., Georgeon, S., Rosset, S., Kapoor, S., Choudhury, J., Dauparas, J., Schellhaas, C., Kozlov, S., Baker, D., Ovchinnikov, S., Vecchio, A. J. & Correia, B. E. (2024). *Nature* **631**, 449–458. 10.1038/s41586-024-07601-y PMC11236705 38898281

[bb82] Grabowski, M., Cooper, D. R., Brzezinski, D., Macnar, J. M., Shabalin, I. G., Cymborowski, M., Otwinowski, Z. & Minor, W. (2021). *Nucl. Instrum. Methods Phys. Res. B* **489**, 30–40. 10.1016/j.nimb.2020.12.016 PMC7886262 33603257

[bb83] Greisman, J. B., Dalton, K. M., Brookner, D. E., Klureza, M. A., Sheehan, C. J., Kim, I.-S., Henning, R. W., Russi, S. & Hekstra, D. R. (2024). *Proc. Natl Acad. Sci. USA* **121**, e2313192121. 10.1073/pnas.2313192121 PMC10907320 38386706

[bb84] Gruhl, T., Weinert, T., Rodrigues, M. J., Milne, C. J., Ortolani, G., Nass, K., Nango, E., Sen, S., Johnson, P. J. M., Cirelli, C., Furrer, A., Mous, S., Skopintsev, P., James, D., Dworkowski, F., Båth, P., Kekilli, D., Ozerov, D., Tanaka, R., Glover, H., Bacellar, C., Brünle, S., Casadei, C. M., Diethelm, A. D., Gashi, D., Gotthard, G., Guixà-González, R., Joti, Y., Kabanova, V., Knopp, G., Lesca, E., Ma, P., Martiel, I., Mühle, J., Owada, S., Pamula, F., Sarabi, D., Tejero, O., Tsai, C.-J., Varma, N., Wach, A., Boutet, S., Tono, K., Nogly, P., Deupi, X., Iwata, S., Neutze, R., Standfuss, J., Schertler, G. & Panneels, V. (2023). *Nature* **615**, 939–944.

[bb85] Gruner, S. M., Tate, M. W. & Eikenberry, E. F. (2002). *Rev. Sci. Instrum.* **73**, 2815–2842.

[bb86] Guo, G., Fuchs, M. R., Shi, W., Skinner, J., Berman, E., Ogata, C. M., Hendrickson, W. A., McSweeney, S. & Liu, Q. (2018). *IUCrJ* **5**, 238–246. 10.1107/S2052252518005389 PMC5929371 29755741

[bb87] Han, H., Round, E., Schubert, R., Gül, Y., Makroczyová, J., Meza, D., Heuser, P., Aepfelbacher, M., Barák, I., Betzel, C., Fromme, P., Kursula, I., Nissen, P., Tereschenko, E., Schulz, J., Uetrecht, C., Ulicný, J., Wilmanns, M., Hajdu, J., Lamzin, V. S. & Lorenzen, K. (2021). *J. Appl. Cryst.* **54**, 7–21. 10.1107/S1600576720013989 PMC7941304 33833637

[bb88] Hara, T., Tanaka, T., Tanabe, T., Maréchal, X.-M., Okada, S. & Kitamura, H. (1998). *J. Synchrotron Rad.* **5**, 403–405. 10.1107/S0909049597015720 15263525

[bb89] Healey, R. D., Basu, S., Humm, A.-S., Leyrat, C., Cong, X., Golebiowski, J., Dupeux, F., Pica, A., Granier, S. & Márquez, J. A. (2021). *Cell Rep. Methods* **1**, 100102. 10.1016/j.crmeth.2021.100102 PMC8545655 34723237

[bb90] Helliwell, J. R. (1992). *Macromolecular Crystallography with Synchrotron Radiation.* Cambridge University Press.

[bb91] Hendrickson, W. A. (2000). *Trends Biochem. Sci.* **25**, 637–643. 10.1016/s0968-0004(00)01721-7 11116192

[bb92] Hendrickson, W. A. (2014). *Q. Rev. Biophys.* **47**, 49–93. 10.1017/S0033583514000018 PMC4128195 24726017

[bb93] Hendrickson, W. A. (2023). *IUCrJ* **10**, 521–543. 10.1107/S2052252523006449 PMC10478523 37668214

[bb94] Hendrickson, W. A., Horton, J. R. & LeMaster, D. M. (1990). *EMBO J.* **9**, 1665–1672. 10.1002/j.1460-2075.1990.tb08287.x PMC551863 2184035

[bb95] Hendrickson, W. A. & Teeter, M. M. (1981). *Nature* **290**, 107–113. 10.1038/290107a0 PMC5536114 28769131

[bb96] Henkel, A. & Oberthür, D. (2024). *Acta Cryst.* D**80**, 563–579. 10.1107/S2059798324005588 PMC11301758 38984902

[bb97] Hennig, M., Ruf, A. & Huber, W. (2012). *Top. Curr. Chem.* **317**, 115–143. 10.1007/128_2011_225 21837555

[bb98] Henrich, B., Bergamaschi, A., Broennimann, C., Dinapoli, R., Eikenberry, E. F., Johnson, I., Kobas, M., Kraft, P., Mozzanica, A. & Schmitt, B. (2009). *Nucl. Instrum. Methods Phys. Res. A* **607**, 247–249.

[bb99] Henzler-Wildman, K. & Kern, D. (2007). *Nature* **450**, 964–972. 10.1038/nature06522 18075575

[bb100] Hilf, R. J. C. & Dutzler, R. (2009). *Nature* **457**, 115–118. 10.1038/nature07461 18987630

[bb101] Hirata, K., Shinzawa-Itoh, K., Yano, N., Takemura, S., Kato, K., Hatanaka, M., Muramoto, K., Kawahara, T., Tsukihara, T., Yamashita, E., Tono, K., Ueno, G., Hikima, T., Murakami, H., Inubushi, Y., Yabashi, M., Ishikawa, T., Yamamoto, M., Ogura, T., Sugimoto, H., Shen, J.-R., Yoshikawa, S. & Ago, H. (2014). *Nat. Methods* **11**, 734–736. 10.1038/nmeth.2962 24813624

[bb102] Hirata, K., Yamashita, K., Ueno, G., Kawano, Y., Hasegawa, K., Kumasaka, T. & Yamamoto, M. (2019). *Acta Cryst.* D**75**, 138–150. 10.1107/S2059798318017795 PMC6400253 30821703

[bb103] Holton, J. & Alber, T. (2004). *Proc. Natl Acad. Sci. USA* **101**, 1537–1542. 10.1073/pnas.0306241101 PMC341770 14752198

[bb104] Holton, J. M. (2009). *J. Synchrotron Rad.* **16**, 133–142.

[bb105] Horrell, S., Kekilli, D., Sen, K., Owen, R. L., Dworkowski, F. S. N., Antonyuk, S. V., Keal, T. W., Yong, C. W., Eady, R. R., Hasnain, S. S., Strange, R. W. & Hough, M. A. (2018). *IUCrJ* **5**, 283–292. 10.1107/S205225251800386X PMC5929374 29755744

[bb106] Huang, C.-Y., Aumonier, S., Engilberge, S., Eris, D., Smith, K. M. L., Leonarski, F., Wojdyla, J. A., Beale, J. H., Buntschu, D., Pauluhn, A., Sharpe, M. E., Metz, A., Olieric, V. & Wang, M. (2022). *Acta Cryst.* D**78**, 964–974. 10.1107/S205979832200612X PMC9344481 35916221

[bb107] Huang, C.-Y., Aumonier, S., Olieric, V. & Wang, M. (2024). *Acta Cryst.* D**80**, 620–628. 10.1107/S2059798324006697 PMC11301757 39052318

[bb108] Huang, C.-Y., Meier, N., Caffrey, M., Wang, M. & Olieric, V. (2020). *J. Appl. Cryst.* **53**, 854–859. 10.1107/S1600576720002897 PMC7312129 32684901

[bb109] Huang, C.-Y., Metz, A., Lange, R., Artico, N., Potot, C., Hazemann, J., Müller, M., Dos Santos, M., Chambovey, A., Ritz, D., Eris, D., Meyer, S., Bourquin, G., Sharpe, M. & Mac Sweeney, A. (2024). *Acta Cryst.* D**80**, 123–136. 10.1107/S2059798324000329 PMC10836397 38289714

[bb110] Huang, C.-Y., Olieric, V., Caffrey, M. & Wang, M. (2020). *Methods Mol. Biol.* **2127**, 293–319. 10.1007/978-1-0716-0373-4_20 32112330

[bb111] Huang, C.-Y., Olieric, V., Howe, N., Warshamanage, R., Weinert, T., Panepucci, E., Vogeley, L., Basu, S., Diederichs, K., Caffrey, M. & Wang, M. (2018). *Commun. Biol.* **1**, 124. 10.1038/s42003-018-0123-6 PMC6123769 30272004

[bb112] Huang, C.-Y., Olieric, V., Ma, P., Howe, N., Vogeley, L., Liu, X., Warshamanage, R., Weinert, T., Panepucci, E., Kobilka, B., Diederichs, K., Wang, M. & Caffrey, M. (2016). *Acta Cryst.* D**72**, 93–112. 10.1107/S2059798315021683 PMC4756617 26894538

[bb113] Huang, C.-Y., Olieric, V., Ma, P., Panepucci, E., Diederichs, K., Wang, M. & Caffrey, M. (2015). *Acta Cryst.* D**71**, 1238–1256. 10.1107/S1399004715005210 PMC4461204 26057665

[bb114] Huang, L., Wang, W., Zhu, Z., Li, Q., Li, M., Zhou, H., Xu, Q., Wen, W., Wang, Q. & Yu, F. (2025). *IUCrJ* **12**, 177–187. 10.1107/S2052252524012247 PMC11878448 39819741

[bb115] Ingold, G., Abela, R., Arrell, C., Beaud, P., Böhler, P., Cammarata, M., Deng, Y., Erny, C., Esposito, V., Flechsig, U., Follath, R., Hauri, C., Johnson, S., Juranic, P., Mancini, G. F., Mankowsky, R., Mozzanica, A., Oggenfuss, R. A., Patterson, B. D., Patthey, L., Pedrini, B., Rittmann, J., Sala, L., Savoini, M., Svetina, C., Zamofing, T., Zerdane, S. & Lemke, H. T. (2019). *J. Synchrotron Rad.* **26**, 874–886. 10.1107/S160057751900331X PMC6510206 31074452

[bb116] Ingold, G., Boege, M., Bulgheroni, W., Keller, A., Krempaski, J., Schulze-Briese, C., Schulz, L., Schmidt, T., Zimoch, D., Hara, T., Tanaka, T. & Kitamura, H. (2007). *AIP Conf. Proc.* **879**, 388–391.

[bb117] Jacquamet, L., Ohana, J., Joly, J., Borel, F., Pirocchi, M., Charrault, P., Bertoni, A., Israel-Gouy, P., Carpentier, P., Kozielski, F., Blot, D. & Ferrer, J.-L. (2004). *Structure* **12**, 1219–1225. 10.1016/j.str.2004.04.019 15242598

[bb118] Jaeger, K., Bruenle, S., Weinert, T., Guba, W., Muehle, J., Miyazaki, T., Weber, M., Furrer, A., Haenggi, N., Tetaz, T., Huang, C.-Y., Mattle, D., Vonach, J.-M., Gast, A., Kuglstatter, A., Rudolph, M. G., Nogly, P., Benz, J., Dawson, R. J. P. & Standfuss, J. (2019). *Cell* **178**, 1222–1230.e10. 10.1016/j.cell.2019.07.028 PMC6709783 31442409

[bb119] Jaho, S., Axford, D., Gu, D.-H., Hough, M. A. & Owen, R. L. (2024). *Methods Enzymol.* **709**, 29–55. 10.1016/bs.mie.2024.10.002 39608947

[bb120] Jinek, M., Jiang, F., Taylor, D. W., Sternberg, S. H., Kaya, E., Ma, E., Anders, C., Hauer, M., Zhou, K., Lin, S., Kaplan, M., Iavarone, A. T., Charpentier, E., Nogales, E. & Doudna, J. A. (2014). *Science* **343**, 1247997. 10.1126/science.1247997 PMC4184034 24505130

[bb121] Juers, D. H., Lovelace, J., Bellamy, H. D., Snell, E. H., Matthews, B. W. & Borgstahl, G. E. O. (2007). *Acta Cryst.* D**63**, 1139–1153. 10.1107/S0907444907045040 18007029

[bb122] Juers, D. H. & Matthews, B. W. (2001). *J. Mol. Biol.* **311**, 851–862. 10.1006/jmbi.2001.4891 11518535

[bb123] Jumper, J., Evans, R., Pritzel, A., Green, T., Figurnov, M., Ronneberger, O., Tunyasuvunakool, K., Bates, R., Žídek, A., Potapenko, A., Bridgland, A., Meyer, C., Kohl, S. A. A., Ballard, A. J., Cowie, A., Romera-Paredes, B., Nikolov, S., Jain, R., Adler, J., Back, T., Petersen, S., Reiman, D., Clancy, E., Zielinski, M., Steinegger, M., Pacholska, M., Berghammer, T., Bodenstein, S., Silver, D., Vinyals, O., Senior, A. W., Kavukcuoglu, K., Kohli, P. & Hassabis, D. (2021). *Nature* **596**, 583–589. 10.1038/s41586-021-03819-2 PMC8371605 34265844

[bb124] Kabsch, W. (2010*a*). *Acta Cryst.* D**66**, 133–144. 10.1107/S0907444909047374 PMC2815666 20124693

[bb125] Kabsch, W. (2010*b*). *Acta Cryst.* D**66**, 125–132. 10.1107/S0907444909047337 PMC2815665 20124692

[bb126] Käck, H. & Sjögren, T. (2025). *J. Synchrotron Rad.* **32**, 294–303. 10.1107/S1600577524012281 PMC11892899 39913304

[bb127] Kaminski, J. W., Vera, L., Stegmann, D., Vering, J., Eris, D., Smith, K. M. L., Huang, C.-Y., Meier, N., Steuber, J., Wang, M., Fritz, G., Wojdyla, J. A. & Sharpe, M. E. (2022). *Acta Cryst.* D**78**, 328–336. 10.1107/S2059798322000705 PMC8900825 35234147

[bb128] Keedy, D. A., Hill, Z. B., Biel, J. T., Kang, E., Rettenmaier, T. J., Brandão-Neto, J., Pearce, N. M., von Delft, F., Wells, J. A. & Fraser, J. S. (2018). *Elife* **7**, e36307. 10.7554/eLife.36307 PMC6039181 29877794

[bb129] Keegan, R. M., Simpkin, A. J. & Rigden, D. J. (2024). *Acta Cryst.* D**80**, 766–779. 10.1107/S2059798324009380 PMC11544426 39360967

[bb130] Kepa, M. W., Tomizaki, T., Sato, Y., Ozerov, D., Sekiguchi, H., Yasuda, N., Aoyama, K., Skopintsev, P., Standfuss, J., Cheng, R., Hennig, M. & Tsujino, S. (2022). *Sci. Rep.* **12**, 5349. 10.1038/s41598-022-09167-z PMC8967846 35354848

[bb131] Kern, J., Yachandra, V. K. & Yano, J. (2015). *Curr. Opin. Struct. Biol.* **34**, 87–98. 10.1016/j.sbi.2015.07.014 PMC4821593 26342144

[bb132] Kitano, H., Matsumura, H., Adachi, H., Murakami, S., Takano, K., Inoue, T., Mori, Y., Doi, M. & Sasaki, T. (2005). *Jpn. J. Appl. Phys.* **44**, L54–L56.

[bb133] Kriminski, S., Caylor, C. L., Nonato, M. C., Finkelstein, K. D. & Thorne, R. E. (2002). *Acta Cryst.* D**58**, 459–471. 10.1107/s0907444902000112 11856832

[bb134] Kriminski, S., Kazmierczak, M. & Thorne, R. E. (2003). *Acta Cryst.* D**59**, 697–708. 10.1107/s0907444903002713 12657789

[bb135] Kühlbrandt, W. (2014). *Science* **343**, 1443–1444. 10.1126/science.1251652 24675944

[bb136] Kupitz, C., Olmos, J. L. Jr, Holl, M., Tremblay, L., Pande, K., Pandey, S., Oberthür, D., Hunter, M., Liang, M., Aquila, A., Tenboer, J., Calvey, G., Katz, A., Chen, Y., Wiedorn, M. O., Knoska, J., Meents, A., Majriani, V., Norwood, T., Poudyal, I., Grant, T., Miller, M. D., Xu, W., Tolstikova, A., Morgan, A., Metz, M., Martin-Garcia, J. M., Zook, J. D., Roy-Chowdhury, S., Coe, J., Nagaratnam, N., Meza, D., Fromme, R., Basu, S., Frank, M., White, T., Barty, A., Bajt, S., Yefanov, O., Chapman, H. N., Zatsepin, N., Nelson, G., Weierstall, U., Spence, J., Schwander, P., Pollack, L., Fromme, P., Ourmazd, A., Phillips, G. N. Jr & Schmidt, M. (2017). *Struct. Dyn.* **4**, 044003. 10.1063/1.4972069 PMC5178802 28083542

[bb137] Lebugle, M., Dworkowski, F., Pauluhn, A., Guzenko, V. A., Romano, L., Meier, N., Marschall, F., Sanchez, D. F., Grolimund, D., Wang, M. & David, C. (2018). *Appl. Opt.* **57**, 9032–9039. 10.1364/AO.57.009032 30461891

[bb138] Lehmann, M. S., Müller, H.-H. & Stuhrmann, H. B. (1993). *Acta Cryst.* D**49**, 308–310. 10.1107/S0907444992011910 15299536

[bb139] Leonarski, F., Brückner, M., Lopez-Cuenca, C., Mozzanica, A., Stadler, H.-C., Matěj, Z., Castellane, A., Mesnet, B., Wojdyla, J. A., Schmitt, B. & Wang, M. (2023). *J. Synchrotron Rad.* **30**, 227–234. 10.1107/S1600577522010268 PMC9814052 36601941

[bb140] Leonarski, F., Mozzanica, A., Brückner, M., Lopez-Cuenca, C., Redford, S., Sala, L., Babic, A., Billich, H., Bunk, O., Schmitt, B. & Wang, M. (2020). *Struct. Dyn.* **7**, 014305. 10.1063/1.5143480 PMC7044001 32128347

[bb141] Leonarski, F., Nan, J., Matej, Z., Bertrand, Q., Furrer, A., Gorgisyan, I., Bjelčić, M., Kepa, M., Glover, H., Hinger, V., Eriksson, T., Cehovin, A., Eguiraun, M., Gasparotto, P., Mozzanica, A., Weinert, T., Gonzalez, A., Standfuss, J., Wang, M., Ursby, T. & Dworkowski, F. (2023). *IUCrJ* **10**, 729–737. 10.1107/S2052252523008618 PMC10619449 37830774

[bb142] Leonarski, F., Redford, S., Mozzanica, A., Lopez-Cuenca, C., Panepucci, E., Nass, K., Ozerov, D., Vera, L., Olieric, V., Buntschu, D., Schneider, R., Tinti, G., Froejdh, E., Diederichs, K., Bunk, O., Schmitt, B. & Wang, M. (2018). *Nat. Methods* **15**, 799–804. 10.1038/s41592-018-0143-7 30275593

[bb143] Leslie, A. G. W. (2006). *Acta Cryst.* D**62**, 48–57. 10.1107/S0907444905039107 16369093

[bb144] Li, H., Huang, C.-Y., Govorunova, E. G., Sineshchekov, O. A., Yi, A., Rothschild, K. J., Wang, M., Zheng, L. & Spudich, J. L. (2021). *Elife* **10**, e65903. 10.7554/eLife.65903 PMC8172240 33998458

[bb145] Liebi, M., Georgiadis, M., Menzel, A., Schneider, P., Kohlbrecher, J., Bunk, O. & Guizar-Sicairos, M. (2015). *Nature* **527**, 349–352. 10.1038/nature16056 26581291

[bb146] Liebschner, D., Yamada, Y., Matsugaki, N., Senda, M. & Senda, T. (2016). *Acta Cryst.* D**72**, 728–741. 10.1107/S2059798316005349 27303793

[bb147] Lima, G. M. A., Talibov, V. O., Jagudin, E., Sele, C., Nyblom, M., Knecht, W., Logan, D. T., Sjögren, T. & Mueller, U. (2020). *Acta Cryst.* D**76**, 771–777. 10.1107/S205979832000889X PMC7397489 32744259

[bb148] Liu, Q., Dahmane, T., Zhang, Z., Assur, Z., Brasch, J., Shapiro, L., Mancia, F. & Hendrickson, W. A. (2012). *Science* **336**, 1033–1037. 10.1126/science.1218753 PMC3769101 22628655

[bb149] Liu, Q., Guo, Y., Chang, Y., Cai, Z., Assur, Z., Mancia, F., Greene, M. I. & Hendrickson, W. A. (2014). *Acta Cryst.* D**70**, 2544–2557. 10.1107/S1399004714013376 PMC4188002 25286840

[bb150] Liu, Q., Liu, Q. & Hendrickson, W. A. (2013). *Acta Cryst.* D**69**, 1314–1332. 10.1107/S0907444913001479 PMC3689535 23793158

[bb151] Liu, Q., Zhang, Z. & Hendrickson, W. A. (2011). *Acta Cryst.* D**67**, 45–59. 10.1107/S0907444910046573 PMC3016016 21206061

[bb152] Liu, Z.-J., Chen, L., Wu, D., Ding, W., Zhang, H., Zhou, W., Fu, Z.-Q. & Wang, B.-C. (2011). *Acta Cryst.* A**67**, 544–549. 10.1107/S0108767311037469 PMC3211246 22011470

[bb153] Lorenz, U. J. (2024). *Curr. Opin. Struct. Biol.* **87**, 102840. 10.1016/j.sbi.2024.102840 38810313

[bb154] Ludeke, A., Andersson, A., Boge, M., Kalantari, B. & Pedrozzi, M. (2006). *Proceedings of the 10th European Particle Accelerator Conference (EPAC2006)*, Edinburgh, UK, 26–30 June 2006, pp. 3424–3426.

[bb155] MacDowell, A. A., Celestre, R. S., Howells, M., McKinney, W., Krupnick, J., Cambie, D., Domning, E. E., Duarte, R. M., Kelez, N., Plate, D. W., Cork, C. W., Earnest, T. N., Dickert, J., Meigs, G., Ralston, C., Holton, J. M., Alber, T., Berger, J. M., Agard, D. A. & Padmore, H. A. (2004). *J. Synchrotron Rad.* **11**, 447–455. 10.1107/S0909049504024835 15496731

[bb156] Madden, J. T., Toth, S. J., Dettmar, C. M., Newman, J. A., Oglesbee, R. A., Hedderich, H. G., Everly, R. M., Becker, M., Ronau, J. A., Buchanan, S. K., Cherezov, V., Morrow, M. E., Xu, S., Ferguson, D., Makarov, O., Das, C., Fischetti, R. & Simpson, G. J. (2013). *J. Synchrotron Rad.* **20**, 531–540. 10.1107/S0909049513007942 PMC3682636 23765294

[bb157] Maestre-Reyna, M., Wang, P.-H., Nango, E., Hosokawa, Y., Saft, M., Furrer, A., Yang, C.-H., Gusti Ngurah Putu, E. P., Wu, W.-J., Emmerich, H.-J., Caramello, N., Franz-Badur, S., Yang, C., Engilberge, S., Wranik, M., Glover, H. L., Weinert, T., Wu, H.-Y., Lee, C.-C., Huang, W.-C., Huang, K.-F., Chang, Y.-K., Liao, J.-H., Weng, J.-H., Gad, W., Chang, C.-W., Pang, A. H., Yang, K.-C., Lin, W.-T., Chang, Y.-C., Gashi, D., Beale, E., Ozerov, D., Nass, K., Knopp, G., Johnson, P. J. M., Cirelli, C., Milne, C., Bacellar, C., Sugahara, M., Owada, S., Joti, Y., Yamashita, A., Tanaka, R., Tanaka, T., Luo, F., Tono, K., Zarzycka, W., Müller, P., Alahmad, M. A., Bezold, F., Fuchs, V., Gnau, P., Kiontke, S., Korf, L., Reithofer, V., Rosner, C. J., Seiler, E. M., Watad, M., Werel, L., Spadaccini, R., Yamamoto, J., Iwata, S., Zhong, D., Standfuss, J., Royant, A., Bessho, Y., Essen, L.-O. & Tsai, M.-D. (2023). *Science* **382**, eadd7795.

[bb158] Martiel, I., Buntschu, D., Meier, N., Gobbo, A., Panepucci, E., Schneider, R., Heimgartner, P., Müller, D., Bühlmann, K., Birri, M., Kaminski, J. W., Leuenberger, J., Oliéric, V., Glettig, W. & Wang, M. (2020). *J. Synchrotron Rad.* **27**, 860–863. 10.1107/S1600577520002416 PMC7285676 32381791

[bb159] Martiel, I., Huang, C.-Y., Villanueva-Perez, P., Panepucci, E., Basu, S., Caffrey, M., Pedrini, B., Bunk, O., Stampanoni, M. & Wang, M. (2020). *IUCrJ* **7**, 1131–1141. 10.1107/S2052252520013238 PMC7642777 33209324

[bb160] Martiel, I., Olieric, V., Caffrey, M. & Wang, M. (2018). *Protein Crystallography*, pp. 1–27. Royal Society of Chemistry.

[bb161] Martin-Garcia, J. M., Zhu, L., Mendez, D., Lee, M.-Y., Chun, E., Li, C., Hu, H., Subramanian, G., Kissick, D., Ogata, C., Henning, R., Ishchenko, A., Dobson, Z., Zhang, S., Weierstall, U., Spence, J. C. H., Fromme, P., Zatsepin, N. A., Fischetti, R. F., Cherezov, V. & Liu, W. (2019). *IUCrJ* **6**, 412–425. 10.1107/S205225251900263X PMC6503920 31098022

[bb162] McLeod, M. J., Barwell, S. A. E., Holyoak, T. & Thorne, R. E. (2025). *Structure* **33**, 924–934.e2. 10.1016/j.str.2025.02.013 40120576

[bb163] Meents, A., Wiedorn, M. O., Srajer, V., Henning, R., Sarrou, I., Bergtholdt, J., Barthelmess, M., Reinke, P. Y. A., Dierksmeyer, D., Tolstikova, A., Schaible, S., Messerschmidt, M., Ogata, C. M., Kissick, D. J., Taft, M. H., Manstein, D. J., Lieske, J., Oberthuer, D., Fischetti, R. F. & Chapman, H. N. (2017). *Nat. Commun.* **8**, 1281. 10.1038/s41467-017-01417-3 PMC5668288 29097720

[bb164] Mehrabi, P., Sung, S., von Stetten, D., Prester, A., Hatton, C. E., Kleine-Döpke, S., Berkes, A., Gore, G., Leimkohl, J.-P., Schikora, H., Kollewe, M., Rohde, H., Wilmanns, M., Tellkamp, F. & Schulz, E. C. (2023). *Nat. Commun.* **14**, 2365. 10.1038/s41467-023-37834-w PMC10130016 37185266

[bb165] Mikolajek, H., Sanchez-Weatherby, J., Sandy, J., Gildea, R. J., Campeotto, I., Cheruvara, H., Clarke, J. D., Foster, T., Fujii, S., Paulsen, I. T., Shah, B. S. & Hough, M. A. (2023). *IUCrJ* **10**, 420–429. 10.1107/S2052252523003810 PMC10324489 37199504

[bb166] Milne, C. J., Schietinger, T., Aiba, M., Alarcon, A., Alex, J., Anghel, A., Arsov, V., Beard, C., Beaud, P., Bettoni, S., Bopp, M., Brands, H., Brönnimann, M., Brunnenkant, I., Calvi, M., Citterio, A., Craievich, P., Csatari Divall, M., Dällenbach, M., D’Amico, M., Dax, A., Deng, Y., Dietrich, A., Dinapoli, R., Divall, E., Dordevic, S., Ebner, S., Erny, C., Fitze, H., Flechsig, U., Follath, R., Frei, F., Gärtner, F., Ganter, R., Garvey, T., Geng, Z., Gorgisyan, I., Gough, C., Hauff, A., Hauri, C. P., Hiller, N., Humar, T., Hunziker, S., Ingold, G., Ischebeck, R., Janousch, M., Juranić, P., Jurcevic, M., Kaiser, M., Kalantari, B., Kalt, R., Keil, B., Kittel, C., Knopp, G., Koprek, W., Lemke, H. T., Lippuner, T., Llorente Sancho, D., Löhl, F., Lopez-Cuenca, C., Märki, F., Marcellini, F., Marinkovic, G., Martiel, I., Menzel, R., Mozzanica, A., Nass, K., Orlandi, G. L., Ozkan Loch, C., Panepucci, E., Paraliev, M., Patterson, B., Pedrini, B., Pedrozzi, M., Pollet, P., Pradervand, C., Prat, E., Radi, P., Raguin, J.-Y., Redford, S., Rehanek, J., Réhault, J., Reiche, S., Ringele, M., Rittmann, J., Rivkin, L., Romann, A., Ruat, M., Ruder, C., Sala, L., Schebacher, L., Schilcher, T., Schlott, V., Schmidt, T., Schmitt, B., Shi, X., Stadler, M., Stingelin, L., Sturzenegger, W., Szlachetko, J., Thattil, D., Treyer, D. M., Trisorio, A., Tron, W., Vetter, S., Vicario, C., Voulot, D., Wang, M., Zamofing, T., Zellweger, C., Zennaro, R., Zimoch, E., Abela, R., Patthey, L. & Braun, H.-H. (2017). *NATO Adv. Sci. Inst. Ser. E Appl. Sci.* **7**, 720.

[bb167] Minor, W., Cymborowski, M., Otwinowski, Z. & Chruszcz, M. (2006). *Acta Cryst.* D**62**, 859–866. 10.1107/S0907444906019949 16855301

[bb168] Moffat, K. & Lattman, E. E. (2023). *Dynamics and kinetics in structural biology.* Nashville: John Wiley & Sons.

[bb169] Monaco, S., Gordon, E., Bowler, M. W., Delagenière, S., Guijarro, M., Spruce, D., Svensson, O., McSweeney, S. M., McCarthy, A. A., Leonard, G. & Nanao, M. H. (2013). *J. Appl. Cryst.* **46**, 804–810. 10.1107/S0021889813006195 PMC3654316 23682196

[bb170] Mous, S., Gotthard, G., Ehrenberg, D., Sen, S., Weinert, T., Johnson, P. J. M., James, D., Nass, K., Furrer, A., Kekilli, D., Ma, P., Brünle, S., Casadei, C. M., Martiel, I., Dworkowski, F., Gashi, D., Skopintsev, P., Wranik, M., Knopp, G., Panepucci, E., Panneels, V., Cirelli, C., Ozerov, D., Schertler, G. F. X., Wang, M., Milne, C., Standfuss, J., Schapiro, I., Heberle, J. & Nogly, P. (2022). *Science* **375**, 845–851. 10.1126/science.abj6663 35113649

[bb171] Mozzanica, A., Andrä, M., Barten, R., Bergamaschi, A., Chiriotti, S., Brückner, M., Dinapoli, R., Fröjdh, E., Greiffenberg, D., Leonarski, F., Lopez-Cuenca, C., Mezza, D., Redford, S., Ruder, C., Schmitt, B., Shi, X., Thattil, D., Tinti, G., Vetter, S. & Zhang, J. (2018). *Synchrotron Radiat. News* **31**(6), 16–20.

[bb172] Muchmore, S. W., Olson, J., Jones, R., Pan, J., Blum, M., Greer, J., Merrick, S. M., Magdalinos, P. & Nienaber, V. L. (2000). *Structure* **8**, R243–R246. 10.1016/s0969-2126(00)00535-9 11188700

[bb173] Mueller, C., Marx, A., Epp, S. W., Zhong, Y., Kuo, A., Balo, A. R., Soman, J., Schotte, F., Lemke, H. T., Owen, R. L., Pai, E. F., Pearson, A. R., Olson, J. S., Anfinrud, P. A., Ernst, O. P. & Dwayne Miller, R. J. (2015). *Struct. Dyn.* **2**, 054302. 10.1063/1.4928706 PMC4711646 26798825

[bb174] Mueller, M., Wang, M. & Schulze-Briese, C. (2012). *Acta Cryst.* D**68**, 42–56. 10.1107/S0907444911049833 PMC3245722 22194332

[bb175] Nam, K. & Wolf-Watz, M. (2023). *Struct. Dyn.* **10**, 014301. 10.1063/4.0000179 PMC9974214 36865927

[bb176] Nannenga, B. L., Shi, D., Leslie, A. G. W. & Gonen, T. (2014). *Nat. Methods* **11**, 927–930. 10.1038/nmeth.3043 PMC4149488 25086503

[bb177] Neubauer, C., Gao, Y.-G., Andersen, K. R., Dunham, C. M., Kelley, A. C., Hentschel, J., Gerdes, K., Ramakrishnan, V. & Brodersen, D. E. (2009). *Cell* **139**, 1084–1095. 10.1016/j.cell.2009.11.015 PMC2807027 20005802

[bb178] Nogly, P., James, D., Wang, D., White, T. A., Zatsepin, N., Shilova, A., Nelson, G., Liu, H., Johansson, L., Heymann, M., Jaeger, K., Metz, M., Wickstrand, C., Wu, W., Båth, P., Berntsen, P., Oberthuer, D., Panneels, V., Cherezov, V., Chapman, H., Schertler, G., Neutze, R., Spence, J., Moraes, I., Burghammer, M., Standfuss, J. & Weierstall, U. (2015). *IUCrJ* **2**, 168–176. 10.1107/S2052252514026487 PMC4392771 25866654

[bb179] Nolting, F., Bostedt, C., Schietinger, T. & Braun, H. (2023). *Eur. Phys. J. Plus* **138**, 126. 10.1140/epjp/s13360-023-03721-y PMC9900202 36779165

[bb180] Ohana, J., Jacquamet, L., Joly, J., Bertoni, A., Taunier, P., Michel, L., Charrault, P., Pirocchi, M., Carpentier, P., Borel, F., Kahn, R. & Ferrer, J.-L. (2004). *J. Appl. Cryst.* **37**, 72–77.

[bb181] O’Hea, J., Burt, M., Fisher, S., Jones, K., McAuley, K., Preece, G. & Williams, M. (2018). *Proceedings of the 16th International Conference on Accelerator and Large Experimental Control Systems (ICALEPCS 2017)*, 8–13 October 2017, Barcelona, Spain, pp. 1919–1922. THPHA200.

[bb182] Olatunji, S., Bowen, K., Huang, C.-Y., Weichert, D., Singh, W., Tikhonova, I. G., Scanlan, E. M., Olieric, V. & Caffrey, M. (2021). *Nat. Commun.* **12**, 4254. 10.1038/s41467-021-24475-0 PMC8275575 34253723

[bb183] Otwinowski, Z. & Minor, W. (1997). *Methods Enzymol.* **276**, 307–326. 10.1016/S0076-6879(97)76066-X 27754618

[bb184] Owen, R. L., Axford, D., Nettleship, J. E., Owens, R. J., Robinson, J. I., Morgan, A. W., Doré, A. S., Lebon, G., Tate, C. G., Fry, E. E., Ren, J., Stuart, D. I. & Evans, G. (2012). *Acta Cryst.* D**68**, 810–818. 10.1107/S0907444912012553 PMC4791751 22751666

[bb185] Owen, R. L., Holton, J. M., Schulze-Briese, C. & Garman, E. F. (2009). *J. Synchrotron Rad.* **16**, 143–151. 10.1107/S0909049508040429 PMC2651761 19240326

[bb186] Owen, R. L., Pearson, A. R., Meents, A., Boehler, P., Thominet, V. & Schulze-Briese, C. (2009). *J. Synchrotron Rad.* **16**, 173–182. 10.1107/S0909049508040120 PMC2651763 19240329

[bb187] Owen, R. L., Rudiño-Piñera, E. & Garman, E. F. (2006). *Proc. Natl Acad. Sci. USA* **103**, 4912–4917. 10.1073/pnas.0600973103 PMC1458769 16549763

[bb188] Pal, A., Debreczeni, J. É., Sevvana, M., Gruene, T., Kahle, B., Zeeck, A. & Sheldrick, G. M. (2008). *Acta Cryst.* D**64**, 985–992. 10.1107/S0907444908022646 18703848

[bb189] Pannu, N. S., Waterreus, W.-J., Skubák, P., Sikharulidze, I., Abrahams, J. P. & de Graaff, R. A. G. (2011). *Acta Cryst.* D**67**, 331–337. 10.1107/S0907444910052224 PMC3069748 21460451

[bb190] Papp, G., Felisaz, F., Sorez, C., Lopez-Marrero, M., Janocha, R., Manjasetty, B., Gobbo, A., Belrhali, H., Bowler, M. W. & Cipriani, F. (2017). *Acta Cryst.* D**73**, 841–851. 10.1107/S2059798317013596 PMC5633909 28994413

[bb191] Pauluhn, A., Pradervand, C., Rossetti, D., Salathe, M. & Schulze-Briese, C. (2011). *J. Synchrotron Rad.* **18**, 595–600. 10.1107/S0909049511011848 PMC3133520 21685676

[bb192] Pearce, N. M., Krojer, T., Bradley, A. R., Collins, P., Nowak, R. P., Talon, R., Marsden, B. D., Kelm, S., Shi, J., Deane, C. M. & von Delft, F. (2017). *Nat. Commun.* **8**, 15123. 10.1038/ncomms15123 PMC5413968 28436492

[bb193] Perrakis, A., Cipriani, F., Castagna, J.-C., Claustre, L., Burghammer, M., Riekel, C. & Cusack, S. (1999). *Acta Cryst.* D**55**, 1765–1770. 10.1107/s0907444999009348 10531527

[bb194] Perrakis, A., Morris, R. & Lamzin, V. S. (1999). *Nat. Struct. Biol.* **6**, 458–463. 10.1038/8263 10331874

[bb195] Pflugrath, J. W. (1999). *Acta Cryst.* D**55**, 1718–1725. 10.1107/s090744499900935x 10531521

[bb196] Phillips, J. C., Wlodawer, A., Yevitz, M. M. & Hodgson, K. O. (1976). *Proc. Natl Acad. Sci. USA* **73**, 128–132. 10.1073/pnas.73.1.128 PMC335853 1061106

[bb197] Pompidor, G., Dworkowski, F. S. N., Thominet, V., Schulze-Briese, C. & Fuchs, M. R. (2013). *J. Synchrotron Rad.* **20**, 765–776. 10.1107/S0909049513016063 PMC3747950 23955041

[bb198] Pradervand, C., Sehr, H., Schulze-Briese, C. & Gobrecht, J. (2004). *Digest of Technical Papers Eurosensors XVIII*, Rome, Italy.

[bb199] Qin, B., Li, Z., Tang, K., Wang, T., Xie, Y., Aumonier, S., Wang, M., Yuan, S. & Cui, S. (2023). *Nat. Commun.* **14**, 3999. 10.1038/s41467-023-39709-6 PMC10326071 37414753

[bb200] Rajendran, C., Dworkowski, F. S. N., Wang, M. & Schulze-Briese, C. (2011). *J. Synchrotron Rad.* **18**, 318–328. 10.1107/S090904951100968X PMC3133521 21525639

[bb201] Rasmussen, B. F., Stock, A. M., Ringe, D. & Petsko, G. A. (1992). *Nature* **357**, 423–424. 10.1038/357423a0 1463484

[bb202] Reardon, S. (2024). *Nature* **635**, 246–248. 10.1038/d41586-024-03595-9 39496959

[bb203] Riley, B. T., Wankowicz, S. A., de Oliveira, S. H. P., van Zundert, G. C. P., Hogan, D. W., Fraser, J. S., Keedy, D. A. & van den Bedem, H. (2021). *Protein Sci.* **30**, 270–285. 10.1002/pro.4001 PMC7737783 33210433

[bb204] Ringe, D. & Petsko, G. A. (2003). *Biophys. Chem.* **105**, 667–680. 10.1016/s0301-4622(03)00096-6 14499926

[bb205] Roedig, P., Duman, R., Sanchez-Weatherby, J., Vartiainen, I., Burkhardt, A., Warmer, M., David, C., Wagner, A. & Meents, A. (2016). *J. Appl. Cryst.* **49**, 968–975. 10.1107/S1600576716006348 PMC4886986 27275143

[bb206] Rossetti, D., Lienert, U., Pradervand, C., Schneider, R., Shi, M., Zelenika, S., Rossat, M., Hignette, O., Rommeveaux, A. & Schulze-Briese, C. (2002). *Proc. SPIE* **4782**, 86–93.

[bb207] Sanchez-Weatherby, J., Sandy, J., Mikolajek, H., Lobley, C. M. C., Mazzorana, M., Kelly, J., Preece, G., Littlewood, R. & Sørensen, T. L.-M. (2019). *J. Synchrotron Rad.* **26**, 291–301. 10.1107/S1600577518015114 PMC6337891 30655497

[bb208] Sauter, N. K., Hattne, J., Brewster, A. S., Echols, N., Zwart, P. H. & Adams, P. D. (2014). *Acta Cryst.* D**70**, 3299–3309. 10.1107/S1399004714024134 PMC4257623 25478847

[bb209] Schneider, T. R. & Sheldrick, G. M. (2002). *Acta Cryst.* D**58**, 1772–1779. 10.1107/s0907444902011678 12351820

[bb210] Schubert, R., Kapis, S., Gicquel, Y., Bourenkov, G., Schneider, T. R., Heymann, M., Betzel, C. & Perbandt, M. (2016). *IUCrJ* **3**, 393–401. 10.1107/S2052252516016304 PMC5094441 27840678

[bb211] Schulze-Briese, C., Heidenreich, G., Auderset, H., Vermeulen, D. & Freund, A. K. (1998). *Proc. SPIE* **3448**, 156–165.

[bb212] Schulze-Briese, C., Ketterer, B., Pradervand, C., Brönnimann, C., David, C., Horisberger, R., Puig-Molina, A. & Graafsma, H. (2001). *Nucl. Instrum. Methods Phys. Res. A* **467–468**, 230–234.

[bb213] Selmer, M., Dunham, C. M., Murphy, F. V., **IV**, Weixlbaumer, A., Petry, S., Kelley, A. C., Weir, J. R. & Ramakrishnan, V. (2006). *Science* **313**, 1935–1942. 10.1126/science.1131127 16959973

[bb214] Sheldrick, G. M. (2002). *Z. Kristallogr. Cryst. Mater.* **217**, 644–650.

[bb215] Sheldrick, G. M. (2010). *Acta Cryst.* D**66**, 479–485. 10.1107/S0907444909038360 PMC2852312 20383001

[bb216] Shin, D., Mukherjee, R., Grewe, D., Bojkova, D., Baek, K., Bhattacharya, A., Schulz, L., Widera, M., Mehdipour, A. R., Tascher, G., Geurink, P. P., Wilhelm, A., van der Heden van Noort, G. J., Ovaa, H., Müller, S., Knobeloch, K.-P., Rajalingam, K., Schulman, B. A., Cinatl, J., Hummer, G., Ciesek, S. & Dikic, I. (2020). *Nature* **587**, 657–662. 10.1038/s41586-020-2601-5 PMC7116779 32726803

[bb217] Sierra, R. G., Weierstall, U., Oberthuer, D., Sugahara, M., Nango, E., Iwata, S. & Meents, A. (2018). *X-ray Free Electron Lasers: A Revolution in Structural Biology*, edited by S. Boutet, P. Fromme & M. S. Hunter. pp. 109–184. Cham: Springer International Publishing.

[bb218] Sikorski, M., Ramilli, M., de Wijn, R., Hinger, V., Mozzanica, A., Schmitt, B., Han, H., Bean, R., Bielecki, J., Bortel, G., Dietze, T., Faigel, G., Kharitonov, K., Kim, C., Koliyadu, J. C. P., Koua, F. H. M., Letrun, R., Lopez, L. M., Reimers, N., Round, A., Sarma, A., Sato, T., Tegze, M. & Turcato, M. (2023). *Front. Phys.* **11**, 1303247.

[bb219] Skopintsev, P., Ehrenberg, D., Weinert, T., James, D., Kar, R. K., Johnson, P. J. M., Ozerov, D., Furrer, A., Martiel, I., Dworkowski, F., Nass, K., Knopp, G., Cirelli, C., Arrell, C., Gashi, D., Mous, S., Wranik, M., Gruhl, T., Kekilli, D., Brünle, S., Deupi, X., Schertler, G. F. X., Benoit, R. M., Panneels, V., Nogly, P., Schapiro, I., Milne, C., Heberle, J. & Standfuss, J. (2020). *Nature* **583**, 314–318. 10.1038/s41586-020-2307-8 32499654

[bb220] Skubák, P. & Pannu, N. S. (2011). *Acta Cryst.* D**67**, 345–354. 10.1107/S0907444911002083 PMC3069750 21460453

[bb221] Smith, J. L., Fischetti, R. F. & Yamamoto, M. (2012). *Curr. Opin. Struct. Biol.* **22**, 602–612. 10.1016/j.sbi.2012.09.001 PMC3478446 23021872

[bb222] Smith, K. M. L., Panepucci, E., Kaminski, J. W., Aumonier, S., Huang, C.-Y., Eris, D., Buntschu, D., Meier, N., Glettig, W., McAuley, K. E., Wang, M., Sharpe, M. E. & Wojdyla, J. A. (2023). *J. Synchrotron Rad.* **30**, 538–545. 10.1107/S1600577523002631 PMC10161886 37042663

[bb223] Soltis, S. M., Cohen, A. E., Deacon, A., Eriksson, T., González, A., McPhillips, S., Chui, H., Dunten, P., Hollenbeck, M., Mathews, I., Miller, M., Moorhead, P., Phizackerley, R. P., Smith, C., Song, J., van dem Bedem, H., Ellis, P., Kuhn, P., McPhillips, T., Sauter, N., Sharp, K., Tsyba, I. & Wolf, G. (2008). *Acta Cryst.* D**64**, 1210–1221. 10.1107/S0907444908030564 PMC2631117 19018097

[bb224] Song, J., Mathew, D., Jacob, S. A., Corbett, L., Moorhead, P. & Soltis, S. M. (2007). *J. Synchrotron Rad.* **14**, 191–195. 10.1107/S0909049507004803 17317920

[bb225] Southworth-Davies, R. J., Medina, M. A., Carmichael, I. & Garman, E. F. (2007). *Structure* **15**, 1531–1541. 10.1016/j.str.2007.10.013 18073104

[bb226] Stachowski, T. R., Vanarotti, M., Seetharaman, J., Lopez, K. & Fischer, M. (2022). *Angew. Chem. Int. Ed.* **61**, e202112919. 10.1002/anie.202112919 PMC9329195 35648650

[bb227] Standfuss, J., Edwards, P. C., D’Antona, A., Fransen, M., Xie, G., Oprian, D. D. & Schertler, G. F. X. (2011). *Nature* **471**, 656–660. 10.1038/nature09795 PMC3715716 21389983

[bb228] Stegmann, D. P., Steuber, J., Fritz, G., Wojdyla, J. A. & Sharpe, M. E. (2023). *Methods Enzymol.* **690**, 235–284. 10.1016/bs.mie.2023.08.005 37858531

[bb229] Stellato, F., Oberthür, D., Liang, M., Bean, R., Gati, C., Yefanov, O., Barty, A., Burkhardt, A., Fischer, P., Galli, L., Kirian, R. A., Meyer, J., Panneerselvam, S., Yoon, C. H., Chervinskii, F., Speller, E., White, T. A., Betzel, C., Meents, A. & Chapman, H. N. (2014). *IUCrJ* **1**, 204–212. 10.1107/S2052252514010070 PMC4107920 25075341

[bb230] Stepanov, S., Hilgart, M., Yoder, D. W., Makarov, O., Becker, M., Sanishvili, R., Ogata, C. M., Venugopalan, N., Aragão, D., Caffrey, M., Smith, J. L. & Fischetti, R. F. (2011). *J. Appl. Cryst.* **44**, 772–778. 10.1107/S0021889811016748 PMC3247932 21808424

[bb231] Strauss, M. G., Westbrook, E. M., Naday, I., Coleman, T. A., Westbrook, M. L., Travis, D. J., Sweet, R. M., Pflugrath, J. W. & Stanton, M. (1990). *Nucl. Instrum. Methods Phys. Res. A* **297**, 275–295.

[bb232] Stuhrmann, S., Bartels, K. S., Braunwarth, W., Doose, R., Dauvergne, F., Gabriel, A., Knöchel, A., Marmotti, M., Stuhrmann, H. B., Trame, C. & Lehmann, M. S. (1997). *J. Synchrotron Rad.* **4**, 298–310. 10.1107/S0909049597009059 16699243

[bb233] Stuhrmann, S., Hütsch, M., Trame, C., Thomas, J. & Stuhrmann, H. B. (1995). *J. Synchrotron Rad.* **2**, 83–86. 10.1107/S0909049594010873 16714793

[bb234] Suga, M., Akita, F., Hirata, K., Ueno, G., Murakami, H., Nakajima, Y., Shimizu, T., Yamashita, K., Yamamoto, M., Ago, H. & Shen, J.-R. (2015). *Nature* **517**, 99–103. 10.1038/nature13991 25470056

[bb235] Sui, S., Wang, Y., Kolewe, K. W., Srajer, V., Henning, R., Schiffman, J. D., Dimitrakopoulos, C. & Perry, S. L. (2016). *Lab Chip* **16**, 3082–3096. 10.1039/c6lc00451b PMC4970872 27241728

[bb236] Sutanto, F., Shaabani, S., Oerlemans, R., Eris, D., Patil, P., Hadian, M., Wang, M., Sharpe, M. E., Groves, M. R. & Dömling, A. (2021). *Angew. Chem. Int. Ed.* **60**, 18231–18239. 10.1002/anie.202105584 PMC8456925 34097796

[bb237] Terwilliger, T. C. (2000). *Acta Cryst.* D**56**, 965–972. 10.1107/S0907444900005072 PMC2792768 10944333

[bb238] Terwilliger, T. C. & Berendzen, J. (1997). *Acta Cryst.* D**53**, 571–579. 10.1107/S0907444997005398 15299888

[bb239] Terwilliger, T. C., Grosse-Kunstleve, R. W., Afonine, P. V., Moriarty, N. W., Zwart, P. H., Hung, L.-W., Read, R. J. & Adams, P. D. (2008). *Acta Cryst.* D**64**, 61–69. 10.1107/S090744490705024X PMC2394820 18094468

[bb240] Thorne, R. E. (2023). *Acta Cryst.* D**79**, 78–94. 10.1107/S2059798322011652 PMC9815097 36601809

[bb241] Tilton, R. F. Jr, Dewan, J. C. & Petsko, G. A. (1992). *Biochemistry* **31**, 2469–2481. 10.1021/bi00124a006 1547232

[bb242] Tolstikova, A., Levantino, M., Yefanov, O., Hennicke, V., Fischer, P., Meyer, J., Mozzanica, A., Redford, S., Crosas, E., Opara, N. L., Barthelmess, M., Lieske, J., Oberthuer, D., Wator, E., Mohacsi, I., Wulff, M., Schmitt, B., Chapman, H. N. & Meents, A. (2019). *IUCrJ* **6**, 927–937. 10.1107/S205225251900914X PMC6760437 31576225

[bb243] Tosstorff, A., Rudolph, M. G., Cole, J. C., Reutlinger, M., Kramer, C., Schaffhauser, H., Nilly, A., Flohr, A. & Kuhn, B. (2022). *J. Comput. Aided Mol. Des.* **36**, 753–765. 10.1007/s10822-022-00478-x 36153472

[bb244] Tsai, M.-D., Wu, W.-J. & Ho, M.-C. (2022). *Annu. Rev. Biophys.* **51**, 19–38. 10.1146/annurev-biophys-100121-075228 34932913

[bb245] Tsujino, S. & Tomizaki, T. (2016). *Sci. Rep.* **6**, 25558. 10.1038/srep25558 PMC4858681 27150272

[bb246] Turk, M. & Baumeister, W. (2020). *FEBS Lett.* **594**, 3243–3261. 10.1002/1873-3468.13948 33020915

[bb247] Ueno, G., Hirose, R., Ida, K., Kumasaka, T. & Yamamoto, M. (2004). *J. Appl. Cryst.* **37**, 867–873.

[bb248] Ursby, T. & Bourgeois, D. (1997). *Acta Cryst.* A**53**, 564–575.

[bb249] Usón, I. & Sheldrick, G. M. (2018). *Acta Cryst.* D**74**, 106–116. 10.1107/S2059798317015121 PMC5947774 29533236

[bb250] Vakili, M., Han, H., Schmidt, C., Wrona, A., Kloos, M., de Diego, I., Dörner, K., Geng, T., Kim, C., Koua, F. H. M., Melo, D. V. M., Rappas, M., Round, A., Round, E., Sikorski, M., Valerio, J., Zhou, T., Lorenzen, K. & Schulz, J. (2023). *J. Appl. Cryst.* **56**, 1038–1045. 10.1107/S1600576723004405 PMC10405586 37555221

[bb251] van den Bedem, H. & Fraser, J. S. (2015). *Nat. Methods* **12**, 307–318. 10.1038/nmeth.3324 PMC4457290 25825836

[bb252] Vonrhein, C., Flensburg, C., Keller, P., Sharff, A., Smart, O., Paciorek, W., Womack, T. & Bricogne, G. (2011). *Acta Cryst.* D**67**, 293–302. 10.1107/S0907444911007773 PMC3069744 21460447

[bb253] Vulpetti, A., Holzer, P., Schmiedeberg, N., Imbach-Weese, P., Pissot-Soldermann, C., Hollingworth, G. J., Radimerski, T., Thoma, C. R., Stachyra, T.-M., Wojtynek, M., Maschlej, M., Chau, S., Schuffenhauer, A., Fernández, C., Schröder, M. & Renatus, M. (2023). *ACS Med. Chem. Lett.* **14**, 949–954. 10.1021/acsmedchemlett.3c00104 PMC10350940 37465299

[bb254] Wagner, A., Diez, J., Schulze–Briese, C. & Schluckebier, G. (2009). *Proteins* **74**, 1018–1027. 10.1002/prot.22213 18767151

[bb255] Wagner, A., Duman, R., Henderson, K. & Mykhaylyk, V. (2016). *Acta Cryst.* D**72**, 430–439. 10.1107/S2059798316001078 PMC4784674 26960130

[bb256] Waltersperger, S., Olieric, V., Pradervand, C., Glettig, W., Salathe, M., Fuchs, M. R., Curtin, A., Wang, X., Ebner, S., Panepucci, E., Weinert, T., Schulze-Briese, C. & Wang, M. (2015). *J. Synchrotron Rad.* **22**, 895–900. 10.1107/S1600577515005354 PMC4489532 26134792

[bb257] Wang, B. C. (1985). *Methods Enzymol.* **115**, 90–112. 10.1016/0076-6879(85)15009-3 4079800

[bb258] Warkentin, M., Badeau, R., Hopkins, J. & Thorne, R. E. (2011). *Acta Cryst.* D**67**, 792–803. 10.1107/S0907444911027600 PMC3169314 21904032

[bb259] Warkentin, M., Badeau, R., Hopkins, J. B., Mulichak, A. M., Keefe, L. J. & Thorne, R. E. (2012). *Acta Cryst.* D**68**, 124–133. 10.1107/S0907444911052085 PMC3266852 22281741

[bb260] Warkentin, M., Hopkins, J. B., Badeau, R., Mulichak, A. M., Keefe, L. J. & Thorne, R. E. (2013). *J. Synchrotron Rad.* **20**, 7–13. 10.1107/S0909049512048303 PMC3526918 23254651

[bb261] Warren, A. J., Axford, D. & Owen, R. L. (2019). *J. Synchrotron Rad.* **26**, 991–997. 10.1107/S1600577519003849 PMC6613110 31274420

[bb262] Wasserman, S. R., Koss, J. W., Sojitra, S. T., Morisco, L. L. & Burley, S. K. (2012). *Trends Pharmacol. Sci.* **33**, 261–267. 10.1016/j.tips.2012.03.009 22521107

[bb263] Weik, M. & Colletier, J.-P. (2010). *Acta Cryst.* D**66**, 437–446. 10.1107/S0907444910002702 PMC2852308 20382997

[bb264] Weinert, T., Olieric, N., Cheng, R., Brünle, S., James, D., Ozerov, D., Gashi, D., Vera, L., Marsh, M., Jaeger, K., Dworkowski, F., Panepucci, E., Basu, S., Skopintsev, P., Doré, A. S., Geng, T., Cooke, R. M., Liang, M., Prota, A. E., Panneels, V., Nogly, P., Ermler, U., Schertler, G., Hennig, M., Steinmetz, M. O., Wang, M. & Standfuss, J. (2017). *Nat. Commun.* **8**, 542. 10.1038/s41467-017-00630-4 PMC5599499 28912485

[bb265] Weinert, T., Olieric, V., Waltersperger, S., Panepucci, E., Chen, L., Zhang, H., Zhou, D., Rose, J., Ebihara, A., Kuramitsu, S., Li, D., Howe, N., Schnapp, G., Pautsch, A., Bargsten, K., Prota, A. E., Surana, P., Kottur, J., Nair, D. T., Basilico, F., Cecatiello, V., Pasqualato, S., Boland, A., Weichenrieder, O., Wang, B.-C., Steinmetz, M. O., Caffrey, M. & Wang, M. (2015). *Nat. Methods* **12**, 131–133. 10.1038/nmeth.3211 25506719

[bb266] Weinert, T., Skopintsev, P., James, D., Dworkowski, F., Panepucci, E., Kekilli, D., Furrer, A., Brünle, S., Mous, S., Ozerov, D., Nogly, P., Wang, M. & Standfuss, J. (2019). *Science* **365**, 61–65. 10.1126/science.aaw8634 31273117

[bb267] White, T. A., Kirian, R. A., Martin, A. V., Aquila, A., Nass, K., Barty, A. & Chapman, H. N. (2012). *J. Appl. Cryst.* **45**, 335–341.

[bb268] Williams, L. J., Thompson, A. J., Dijkstal, P., Appleby, M., Assmann, G., Dworkowski, F. S. N., Hiller, N., Huang, C.-Y., Mason, T., Perrett, S., Prat, E., Voulot, D., Pedrini, B., Beale, J. H., Hough, M. A., Worrall, J. A. R. & Owen, R. L. (2025). *IUCrJ* **12**, 358–371. 10.1107/S2052252525002660 PMC12044858 40227256

[bb269] Williamson, A. R. (2000). *Nat. Struct. Biol.* **7**, 953. 10.1038/80726 11103997

[bb270] Willmott, P. R. & Braun, H. (2024). *Synchrotron Radiat. News* **37**(1), 24–32.

[bb271] Winter, G. (2010). *J. Appl. Cryst.* **43**, 186–190.

[bb272] Winter, G., Gildea, R. J., Paterson, N., Beale, J., Gerstel, M., Axford, D., Vollmar, M., McAuley, K. E., Owen, R. L., Flaig, R., Ashton, A. W. & Hall, D. R. (2019). *Acta Cryst.* D**75**, 242–261. 10.1107/S2059798319003528 PMC6450062 30950396

[bb273] Wojdyla, J. A., Panepucci, E., Martiel, I., Ebner, S., Huang, C.-Y., Caffrey, M., Bunk, O. & Wang, M. (2016). *J. Appl. Cryst.* **49**, 944–952. 10.1107/S1600576716006233 PMC4886984 27275141

[bb274] Woldeyes, R. A., Sivak, D. A. & Fraser, J. S. (2014). *Curr. Opin. Struct. Biol.* **28**, 56–62. 10.1016/j.sbi.2014.07.005 PMC4253534 25113271

[bb275] Wolff, A. M., Nango, E., Young, I. D., Brewster, A. S., Kubo, M., Nomura, T., Sugahara, M., Owada, S., Barad, B. A., Ito, K., Bhowmick, A., Carbajo, S., Hino, T., Holton, J. M., Im, D., O’Riordan, L. J., Tanaka, T., Tanaka, R., Sierra, R. G., Yumoto, F., Tono, K., Iwata, S., Sauter, N. K., Fraser, J. S. & Thompson, M. C. (2023). *Nat. Chem.* **15**, 1549–1558. 10.1038/s41557-023-01329-4 PMC10624634 37723259

[bb276] Wollenhaupt, J., Barthel, T., Lima, G. M. A., Metz, A., Wallacher, D., Jagudin, E., Huschmann, F. U., Hauß, T., Feiler, C. G., Gerlach, M., Hellmig, M., Förster, R., Steffien, M., Heine, A., Klebe, G., Mueller, U. & Weiss, M. S. (2021). *J. Vis. Exp.* **169**, e62208. 10.3791/62208 33749678

[bb277] Wranik, M., Weinert, T., Slavov, C., Masini, T., Furrer, A., Gaillard, N., Gioia, D., Ferrarotti, M., James, D., Glover, H., Carrillo, M., Kekilli, D., Stipp, R., Skopintsev, P., Brünle, S., Mühlethaler, T., Beale, J., Gashi, D., Nass, K., Ozerov, D., Johnson, P. J. M., Cirelli, C., Bacellar, C., Braun, M., Wang, M., Dworkowski, F., Milne, C., Cavalli, A., Wachtveitl, J., Steinmetz, M. O. & Standfuss, J. (2023). *Nat. Commun.* **14**, 903. 10.1038/s41467-023-36481-5 PMC9936131 36807348

[bb278] Yabukarski, F., Doukov, T., Pinney, M. M., Biel, J. T., Fraser, J. S. & Herschlag, D. (2022). *Sci. Adv.* **8**, eabn7738. 10.1126/sciadv.abn7738 PMC9565801 36240280

[bb279] Yamano, T., Nishimasu, H., Zetsche, B., Hirano, H., Slaymaker, I. M., Li, Y., Fedorova, I., Nakane, T., Makarova, K. S., Koonin, E. V., Ishitani, R., Zhang, F. & Nureki, O. (2016). *Cell* **165**, 949–962. 10.1016/j.cell.2016.04.003 PMC4899970 27114038

[bb280] Yao, Y., Zhou, T., Färber, R., Grossner, U., Floudas, G. & Mezzenga, R. (2021). *Nat. Nanotechnol.* **16**, 802–810. 10.1038/s41565-021-00893-5 33941918

[bb281] Zander, U., Bourenkov, G., Popov, A. N., de Sanctis, D., Svensson, O., McCarthy, A. A., Round, E., Gordeliy, V., Mueller-Dieckmann, C. & Leonard, G. A. (2015). *Acta Cryst.* D**71**, 2328–2343. 10.1107/S1399004715017927 PMC4631482 26527148

[bb282] Zarrine-Afsar, A., Barends, T. R. M., Müller, C., Fuchs, M. R., Lomb, L., Schlichting, I. & Miller, R. J. D. (2012). *Acta Cryst.* D**68**, 321–323. 10.1107/S0907444911055296 22349234

[bb283] Zhang, K. Y. J. & Main, P. (1990). *Acta Cryst.* A**46**, 41–46.

[bb284] Zhang, Z., Sauter, N. K., van den Bedem, H., Snell, G. & Deacon, A. M. (2006). *J. Appl. Cryst.* **39**, 112–119.

[bb285] Zheng, H., Hou, J., Zimmerman, M. D., Wlodawer, A. & Minor, W. (2014). *Exp. Opin. Drug. Discov.* **9**, 125–137. 10.1517/17460441.2014.872623 PMC4106240 24372145

