# Supplementary material for: Evolution of macromolecular crystallography beamlines at the Swiss Light Source and SwissFEL
Source: J Synchrotron Radiat. 2025 Jul 14;32(Pt 5):1162–83. doi: 10.1107/S1600577525005016 (PMC12416430; doi:10.1107/S1600577525005016)
Supplement: Supplementary file 1 [file s-32-01162-sup1.pdf]

JOURNAL OF SYNCHROTRON RADIATION

Volume 32 (2025)

Supporting information for article:

Evolution of macromolecular crystallography beamlines at the Swiss Light Source and SwissFEL

Meitian Wang

Supplementary Table 1. X-ray optics specifications

	X06SA-PXI			X10SA-PXII			X06DA-PXIII	
	SLS		SLS 2.0	SLS		SLS 2.0	SLS	SLS 2.0
Start year	2001	2012	2025	2004	2017	2025	2008	2025
Machine energy (GeV)	2.4	2.4	2.7	2.4	2.4	2.7	2.4	2.7
Source	U19, 1.9 m	U19, 1.9 m	U17, 3 m	U19, 1.9m	U19, 1.9 m	U17, 3 m	SB, 2.7 T	2.1 T
Energy (keV)	6 - 17.5	6 - 17.5	5 - 30	6 - 20	6 - 20	5 - 30	5 - 17.5	4 - 15
Monochromator[1]	V-double crystal Si(111) 19.65 m	V-double crystal Si(111) 19.65 m	H-double crystal Si(111), 18.2 m Double multilayer 17.135 m	V-double crystal Si(111) 19 m	V-double crystal Si(111) 19 m	H-double crystal Si(111) 1st-xtal 18.2 m	V-double channel-cut Si(111) 12.9, 13.3 m	V-double channel-cut Si(111) 13.4, 13.8 m
Primary focusing[2]	Sagittal bender 19.65 m	Sagittal bender 19.65 m	V-KB mirror (3 mrad) 23.75 m	Sagittal bender 19 m	Sagittal bender 19 m	V-KB mirror (3 mrad) 24.15 m	V-collimating mirror (3.75 mrad) 7 m	Toroidal mirror (4.5 mrad) 6.9 m
	Vertical focusing mirror (3.75 mrad) 20.5 m	Vertical focusing mirror (3.75 mrad) 20.5 m	H-KB mirror (3 mrad) 24.5 m	Vertical focusing mirror (3.75 mrad) 19.9 m	Vertical focusing mirror (3.75 mrad) 19.9 m	H-KM mirror (3 mrad) 24.9 m	Toroidal mirror (3.75 mrad) 14 m	-
Secondary source	-	24.5 - 26.5 m	X-ray chopper 22.550 m	-	22.5 m	22.239 m	-	Horizontal 20.7 m
Secondary focusing[3]	-	V-KB mirror (3.75 mrad)	-	-	V-1D Kinoform lenses	2D Be-CRLs 19.5 - 20.2 m	-	V-KB mirror (4.5 mrad)

		27.22 m			25.08 m			22.95 m
	-	H-KB mirror (3.75 mrad) 27.5 m	-	-	H-1D Kinoform lenses 25.25 m	2D Be-CRLs 19.6 - 20.3 m	-	H-KB mirror (4.5 mrad) 23.7 m
Sample position	22.5 m, 28 m	28 m	26.5 m	23.75 m	25.6 m	25.6 m	21 m	25.2 m

1. V and H refer to vertical and horizontal monochromator configurations. For SLS Si-monochromator, the fixed-exit is achieved by moving the first crystal along the beam direction. The distance-to-source is for the second crystal. For SLS 2.0 monochromators, two crystals are mounted around one Bragg rotation axis. The distance-to-source is for the Bragg axis.

2. V and H refer to focusing in vertical and horizontal directions, respectively.

3. SLS 2.0 X10SA-PXII 2D Beryllium Compound Refractive Lenses (CRLs) are used to alter the focus point of the primary focusing of KB mirrors

Supplementary Figure 1. Statistics of PDB structure depositions and publications from PXI/II/III at the SLS

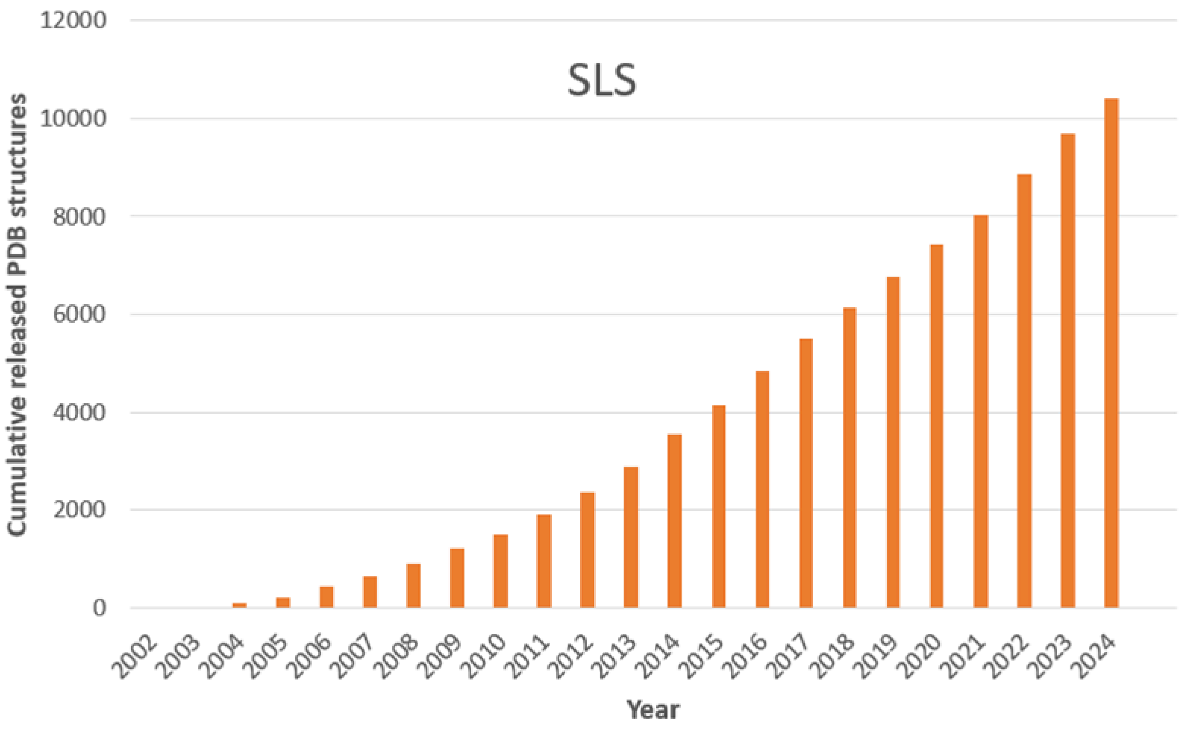

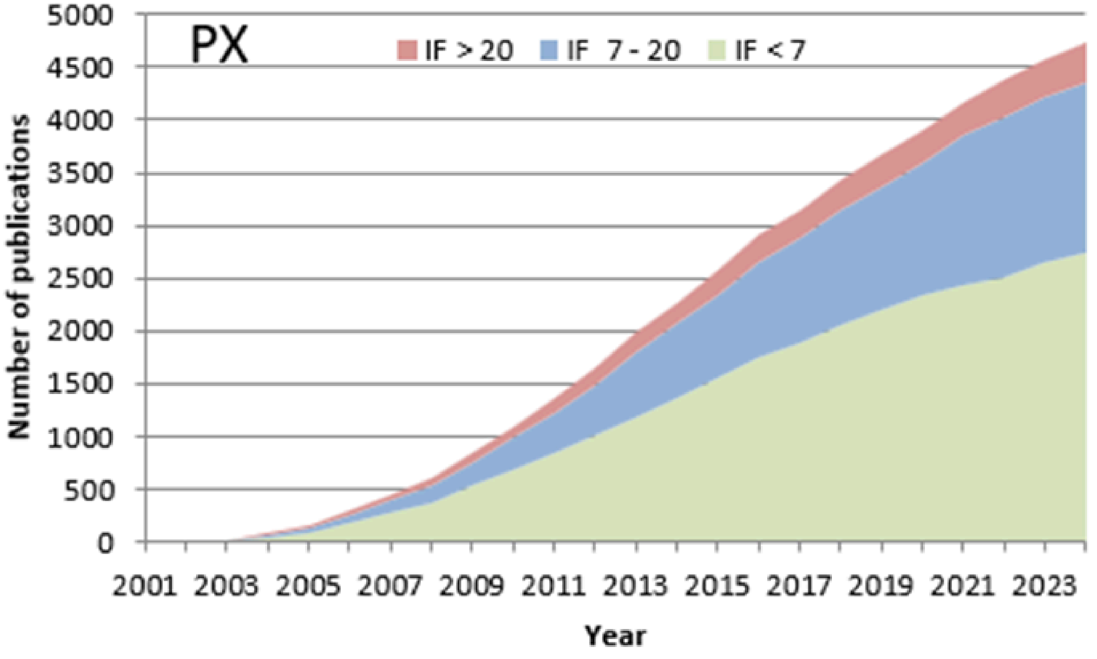

Supplementary Figure 2. X06SA-PXI upgrade in 2012 with two-stage focusing and a variable secondary source position to control the beam size and divergence to match crystal size and applications. The fluxes are for 12.4 keV X-rays.

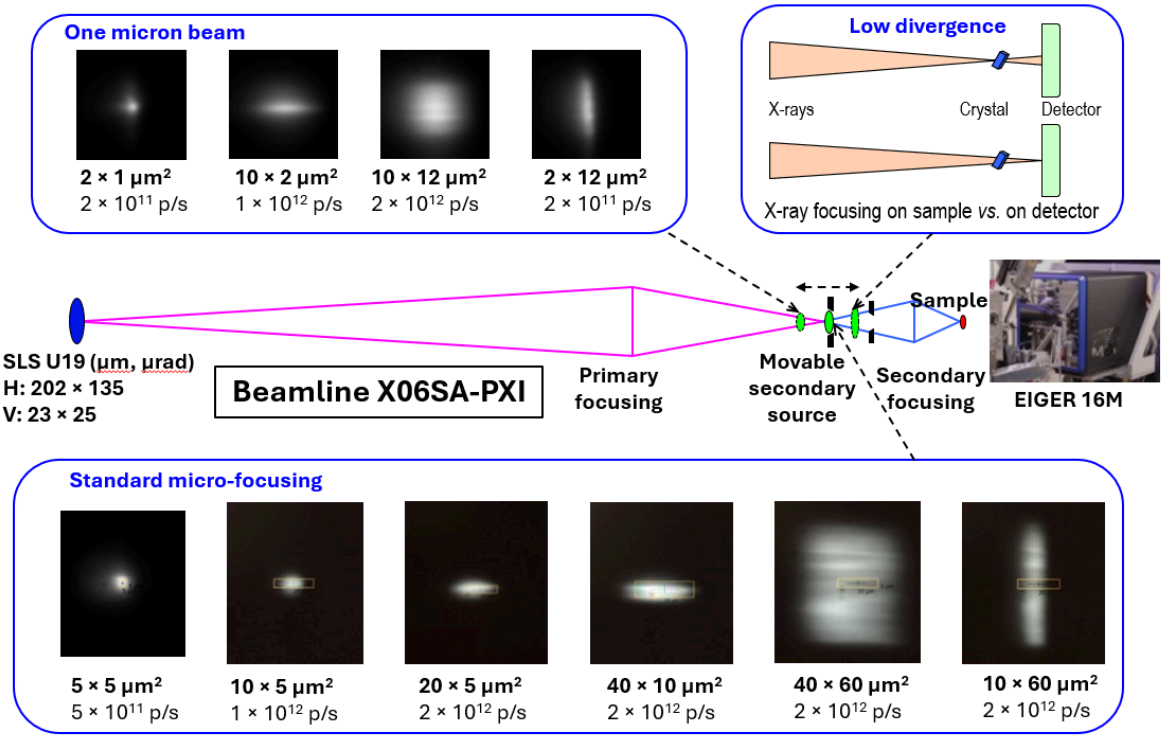

Supplementary Figure 3. An inside view of the X06DA-PXIII mini-hutch showing two access windows for manual mounting (right) and puck loading (left).

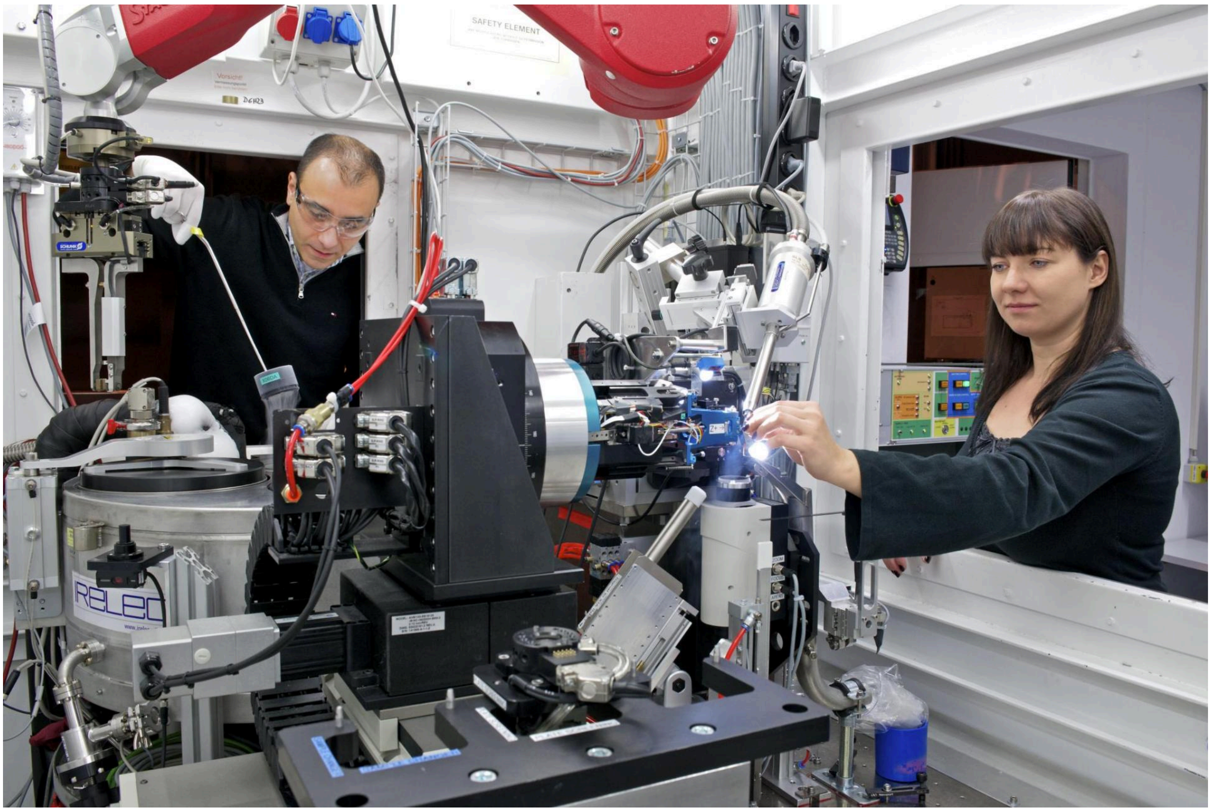

Supplementary Figure 4. The first PILATUS 6M at X06SA-PXI and a representative diffraction image from 30S ribosomal subunit, showing the resolving power of the detector.

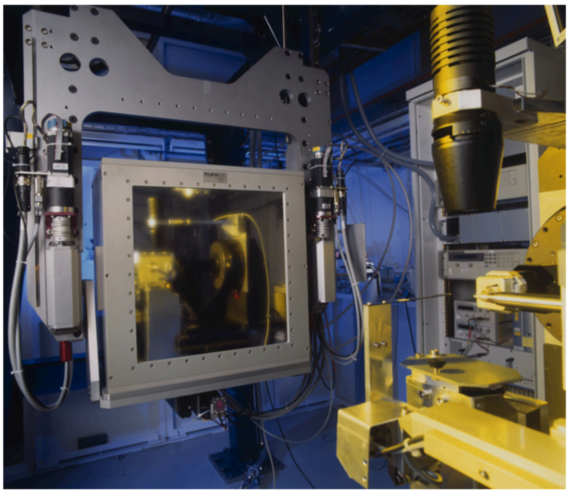

2007 X06SA-PXI PILATUS 6M, PSI detector group

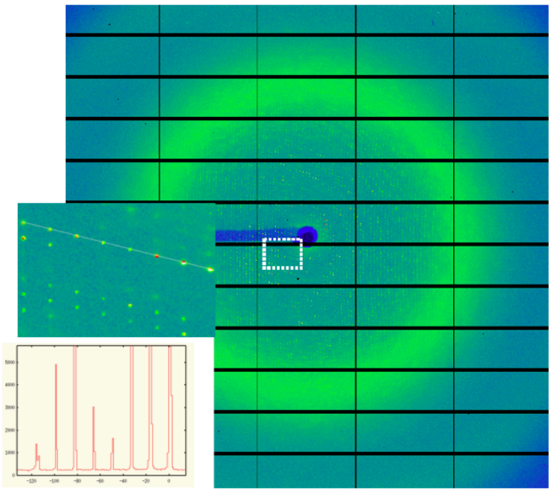

30S ribosomal subunit, Ramakrishnan Lab (MRC, UK)
